# SEC23A rescues SEC23B-deficient congenital dyserythropoietic anemia type II

**DOI:** 10.1126/sciadv.abj5293

**Published:** 2021-11-24

**Authors:** Richard King, Zesen Lin, Ginette Balbin-Cuesta, Gregg Myers, Ann Friedman, Guojing Zhu, Beth McGee, Thomas L. Saunders, Ryo Kurita, Yukio Nakamura, James Douglas Engel, Pavan Reddy, Rami Khoriaty

**Affiliations:** 1 Department of Internal Medicine, University of Michigan, Ann Arbor, MI, USA.; 2 University of Michigan Rogel Cancer Center, Ann Arbor, MI, USA.; 3 Department of Pharmacology, University of Michigan, Ann Arbor, MI, USA.; 4 Cellular and Molecular Biology Program, University of Michigan, Ann Arbor, MI, USA.; 5 Medical Scientist Training Program, University of Michigan, Ann Arbor, MI, USA.; 6 Department of Cell and Developmental Biology, University of Michigan, Ann Arbor, MI, USA.; 7 Life Sciences Institute, University of Michigan, Ann Arbor, MI, USA.; 8 Transgenic Animal Model Core, University of Michigan, Ann Arbor, MI, USA.; 9 Department of Research and Development, Central Blood Institute, Blood Service Headquarters, Japanese Red Cross Society, Tokyo, Japan.; 10 Cell Engineering Division, RIKEN BioResource Research Center, Ibaraki, Japan.

## Abstract

Congenital dyserythropoietic anemia type II (CDAII) results from loss-of-function mutations in *SEC23B*. In contrast to humans, SEC23B-deficient mice deletion do not exhibit CDAII but die perinatally with pancreatic degeneration. Here, we demonstrate that expression of the full SEC23A protein (the SEC23B paralog) from the endogenous regulatory elements of *Sec23b* completely rescues the SEC23B-deficient mouse phenotype. Consistent with these data, while mice with erythroid-specific deletion of either *Sec23a* or *Sec23b* do not exhibit CDAII, we now show that mice with erythroid-specific deletion of all four *Sec23* alleles die in mid-embryogenesis with features of CDAII and that mice with deletion of three *Sec23* alleles exhibit a milder erythroid defect. To test whether the functional overlap between the *SEC23* paralogs is conserved in human erythroid cells, we generated SEC23B-deficient HUDEP-2 cells. Upon differentiation, these cells exhibited features of CDAII, which were rescued by increased expression of SEC23A, suggesting a novel therapeutic strategy for CDAII.

## Teaser

The COPII component SEC23A overlaps functionally with its paralogous protein and rescues the SEC23B-deficient CDAII defect.

## INTRODUCTION

The congenital dyserythropoietic anemias (CDAs) are a group of hereditary disorders resulting from defects in late erythroid maturation (*1*). CDAII, the most common CDA (*2*, *3*), is characterized by ineffective erythropoiesis, anemia of variable severity (*1*, *4*–*6*), and increased percentage of bone marrow (BM) binucleated erythroid precursors. Additional characteristic features of CDAII include hypoglycosylation of the erythrocyte plasma membrane protein band 3 (*7*) and residual endoplasmic reticulum (ER) in red blood cells (RBCs) resulting in a double plasma membrane appearance by electron microscopy (*8*). CDAII is an autosomal recessive disease resulting from loss-of-function mutations in *SEC23B* (*4*).

Although the genetic defect underlying CDAII was identified more than a decade ago, the mechanism by which SEC23B deficiency results in ineffective erythropoiesis remains unknown, and there are no well-defined therapies for CDAII. To study the role of SEC23B in erythropoiesis, we previously generated mice with germline deletion of *Sec23b*. Unexpectedly, these mice died in the perinatal period exhibiting profound degeneration of their pancreatic tissues (*9*–*11*). The perinatal lethality of these mice precluded evaluation of the adult SEC23B-deficient hematopoietic compartment. To examine the impact of SEC23B deficiency on adult murine hematopoiesis, we next generated mice deficient for SEC23B in the erythroid or panhematopoietic compartments. These tissue-specific knock-out mice did not exhibit anemia or other CDAII characteristics (*12*), an unexpected and unanticipated result. Similarly, lethally irradiated wild-type (WT) mice transplanted with *Sec23b*-deleted fetal liver cells failed to exhibit a CDAII phenotype (*12*).

SEC23 is a component of coat complex II (COPII) vesicles, which traffic ~7000 mammalian proteins from the ER to the Golgi apparatus (*13*, *14*). The mammalian genome encodes multiple paralogs for most components of the COPII coat, including two paralogs for SEC23, SEC23A, and SEC23B (*15*), which share ~85% amino acid sequence identity (*16*). Previous studies from our laboratory demonstrated rescue of the SEC23B-deficient murine phenotype by expression of a chimeric SEC23B-A protein (consisting of the first 122 amino acids of SEC23B and amino acids 123 to 765 of SEC23A) from the endogenous genomic locus of *Sec23b* (*17*). However, other reports suggested unique functions for SEC23A and SEC23B, with each paralog facilitating selective and tissue-specific cargo capture and secretion (*18*–*25*), therefore raising the possibility that the rescue by the SEC23B-A allele resulted from contribution of the residual SEC23B N-terminal tail. In addition, and pertinent to CDAII, it remains unknown whether SEC23A and SEC23B overlap in function in erythroid cells.

Here, we address several outstanding questions in the field. We first generated mice expressing full-length *Sec23a* under the control of *Sec23b* regulatory elements and demonstrated complete phenotypic rescue of the SEC23B-deficient phenotype, excluding a critical contribution from the residual SEC23B N-terminal tail in our previously reported mice. In addition, analysis of the erythroid compartments of mice with erythroid-specific deletion of all combinations of the four *Sec23* alleles established the first murine model of human CDAII and demonstrated an inverse correlation between the total SEC23 level in erythroid cells and the severity of the erythroid phenotype. Furthermore, we generated human umbilical cord blood–derived erythroid progenitor-2 (HUDEP-2) cell lines that are deficient in SEC23B; these cell lines exhibited features of human CDAII, which were equally rescued by expression of either SEC23A or SEC23B, demonstrating that the functional equivalence of SEC23A and SEC23B is also reflected in human erythroid cells. Moreover, we showed that increasing SEC23A levels using CRISPRa rescues the SEC23B-deficient erythroid defect observed in HUDEP-2 cells. Together, these results suggest that increasing the expression of the unaffected *SEC23A* gene may prove a novel therapeutic strategy for CDAII.

## RESULTS

### Generation of mice expressing full-length *Sec23a* under the control of *Sec23b* regulatory elements

**Fig. 1.**
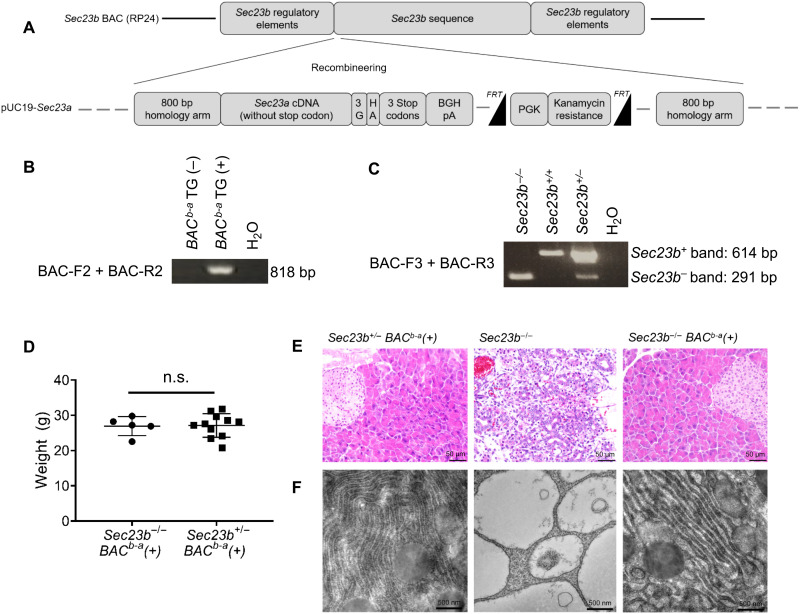
SEC23A rescues the mortality and pancreatic degeneration of SEC23B-deficient mice. (**A**) The coding sequence of *Sec23a* (*Sec23a* cDNA) was recombineered into the *Sec23b* BAC RP24, which contains the entire *Sec23b* gene and its cis-regulatory elements. The resulting *Sec23^b-a^* BAC (*BAC^b-a^*) expresses *Sec23a* from the regulatory elements of *Sec23b* (not drawn to scale). (**B**) PCR genotyping performed on genomic DNA extracted from mice tail biopsies shows the expected band size for the presence of the *BAC^b-a^* transgene. (**C**) To distinguish the *Sec23b^−/−^ BAC^b-a^(+)* from the *Sec23b^+/−^ BAC^b-a^(+)* genotypes, RT-PCR was performed on RNA isolated from tail biopsies using a forward and reverse primer located in *Sec23b* exons 2 and 7, respectively. The *Sec23b^−^* allele has been previously shown to be dysfunctional. (**D**) *Sec23b^−/−^ BAC^b-a^(+)* mice (*n* = 5) exhibit normal weights, indistinguishable from those of *Sec23b^+/−^ BAC^b-a^(+)* littermate control mice (*n* = 11). (**E**) The pancreatic degeneration (H&E staining) and (**F**) dilated ER (electron microscopy) observed in *Sec23b^−/−^* pancreas tissues are rescued by the *SEC23^b-a^* BAC. BAC, bacterial artificial chromosome; bp, base pair; G, glycine; HA, hemagglutinin; BGH pA, bovine growth hormone polyadenylation; PGK, phosphoglycerate kinase promoter. Two-sided, unpaired *t* tests were used to compare means. *P* values not significant (n.s.) if not marked. Data represent means ± SD.

To determine whether SEC23A overlaps in function with SEC23B in vivo, we generated mice that express the full-lengh *Sec23a* under the control of *Sec23b* regulatory elements. To generate these mice, we obtained a ~170-kb mouse bacterial artificial chromosome (BAC) that spans the *Sec23b* gene and that contains the key regulatory sequences for *Sec23b*, as we previously described (*10*). We inserted the *Sec23a* coding sequence at the translational start of *Sec23b* by homologous recombination (Fig. 1A). Correct insertion of the *Sec23a* coding sequence in the BAC and sequence integrity of both the inserted DNA and the insert junctions were confirmed by Sanger sequencing. This recombineered BAC, referred to as *Sec23^b-a^* BAC, expresses SEC23A under the regulatory elements of *Sec23b*. Following pronuclear injection of the *Sec23^b-a^* BAC in C57BL/6J oocytes fertilized with sperm from *Sec23b^+/−^* male mice and implantation of the injected zygotes into pseudopregnant females, germline transmission of the *Sec23^b-a^* BAC transgene was obtained.

### SEC23A rescues the mortality and pancreatic degeneration of SEC23B-deficient mice

To determine whether the recombineered *Sec23^b-a^* BAC expressing SEC23A from the *Sec23b* locus rescues the perinatal lethality of SEC23B-deficient mice, mice that are heterozygous for the *Sec23b* null allele and hemizygous for the *Sec23^b-a^* BAC (*Sec23b^+/−^ Sec23^b-a^* BAC^+^ mice) were crossed to *Sec23b^+/−^* mice. The latter cross generated *Sec23b^−/−^ Sec23^b-a^* BAC^+^ mice at the expected Mendelian ratio at weaning (Table 1A and Fig. 1, B and C).

**Table 1.** Mouse crosses. (**A**) *Sec23b^−/−^* mice expressing *Sec23a* from the *Sec23b* locus (*BAC^b-a^*) are observed at the expected ratios at weaning. (**B** and **C**) Mice with erythroid Sec23a deletion are observed at the expected ratios at weaning. (**D** and **E**) Mice with combined *Sec23* (*Sec23a* + *Sec23b*) deletion in the erythroid compartment are not observed at weaning, (**F**) but they are not underrepresented at E12.5. (**G** to **I**) Mice with *Sec23a* haploinsufficiency and *Sec23b* deletion are not observed at weaning (except for one mouse, which died before assessing excision efficiency); however, (**J** to **L**) these mice were not underrepresented at E14.5. (**M** to **O**) Mice with *Sec23a* deletion and *Sec23b* haploinsufficiency are observed at the expected ratios at weaning. *P* value was calculated by chi-square analysis for genotype of interest in each cross (indicated by #) versus other genotypes.

**A. *Sec23b*^*+/−*^ *BAC*^*b-a*^ *(+)* × *Sec23b*^*+/−*^**						
**Genotypes**	***Sec23b*^−/−^ *BAC*^*b-a*^ (+) #**	**Others**				***P* value^a^**
**Expected ratios**	**14.3%**	**85.7%**				
Observed at weaning % (*n* = 32)	18.8% (6)	81.2% (26)				0.47
**B. *Sec23a*^*+/−*^ *EpoR*-Cre (+) × *Sec23a*^*fl/fl*^**						
**Genotypes**	***Sec23a* ^−/*fl*^** ***EpoR*-Cre (+) #**	***Sec23a* ^*+/fl*^** ***EpoR*-Cre (+)**	**Others**			***P* value^b^**
**Expected ratios**	**25%**	**25%**	**50%**			
Observed at weaning % (*n* = 65)	30.8% (20)	29.2% (19)	40.0% (26)			0.28
**C. *Sec23a*^−/*fl*^ *EpoR*-Cre (+) × *Sec23a*^*fl/fl*^**						
**Genotypes**	***Sec23a* ^−/*fl*^** ***EpoR*-Cre (+) #**	***Sec23a* ^*fl/fl*^** ***EpoR*-Cre (+) #**	**Others**			***P* value^c^**
**Expected ratios**	**25%**	**25%**	**50%**			
Observed at weaning % (*n* = 62)	33.9% (21)	19.3% (12)	46.8% (29)			0.61
**D. *Sec23a*^*fl/fl*^ *Sec23b*^*+/fl*^ *EpoR*-Cre (+) × *Sec23a*^*fl/fl*^ *Sec23b*^*fl/fl*^**						
**Genotypes**	***Sec23a*^*fl/fl*^ *Sec23b*^*fl/fl*^** ***EpoR*-Cre (+) #**	**Others**				***P* value^d^**
**Expected ratios**	**25%**	**75%**				
Observed at weaning % (*n* = 125)	0% (0)	100% (125)				<0.0001
**E. *Sec23a*^−/*fl*^ *Sec23b*^+/−^ *EpoR*-Cre (+) × *Sec23a*^*fl/fl*^ *Sec23b*^*fl/fl*^**						
**Genotypes**	***Sec23a*^*fl/fl*^ *Sec23b*^−/*fl*^** ***EpoR*-Cre (+) #**	***Sec23a*^−/*fl*^ *Sec23b*^−/*fl*^** ***EpoR*-Cre (+) #**	**Others**			***P* value^e^**
**Expected ratios**	**12.5%**	**12.5%**	**75%**			
Observed at weaning % (*n* = 67)	0% (0)	0% (0)	100% (67)			<0.0001
**F. *Sec23a*^*fl/fl*^ *Sec23b*^*+/fl*^ *EpoR*-Cre (+) × *Sec23a*^*fl/fl*^ *Sec23b*^*fl/fl*^**						
**Genotypes**	***Sec23a*^*fl/fl*^ *Sec23b*^*fl/fl*^** ***EpoR*-Cre (+) #**	**Others**				***P* value^f^**
**Expected ratios**	**25%**	**75%**				
Observed at E12.5% (*n* = 15)	46.7% (7)	53.3% (8)				0.0526
**G. *Sec23b*^−/*fl*^ *EpoR*-Cre (+) × *Sec23a*^+/−^ *Sec23b*^*+/−*^**						
**Genotypes**	***Sec23a*^*+/−*^ *Sec23b*^−/*fl*^** ***EpoR*-Cre (+) #**	**Others**				***P* value^g^**
**Expected ratios**	**8.3%**	**91.7%**				
Observed at weaning % (*n* = 94)	0% (0)	100% (94)				0.0035
**H. *Sec23b*^−/*fl*^ *EpoR*-Cre (+) × *Sec23a*^*fl/fl*^ *Sec23b*^*fl/fl*^**						
**Genotypes**	***Sec23a*^*+/fl*^ *Sec23b*^*fl/fl*^** ***EpoR*-Cre (+) #**	***Sec23a*^*+/fl*^ *Sec23b*^−/*fl*^** ***EpoR*-Cre (+) #**	**Others**			***P* value^h^**
**Expected ratios**	**25%**	**25%**	**50%**			
Observed at weaning % (*n* = 45)	4.4% (2)	2.2% (1)	93.3% (42)			<0.0001
**I. *Sec23b*^*fl/fl*^ *EpoR-*Cre (+) × *Sec23a*^+/−^ *Sec23b*^*+/−*^**						
**Genotypes**	***Sec23a*^*+/−*^ *Sec23b*^−/*fl*^** ***EpoR*-Cre (+) #**	**Others**				***P* value^i^**
**Expected ratios**	**12.5%**	**87.5%**				
Observed at weaning % (*n* = 16)	0% (0)	100% (16)				0.13
**J. *Sec23b*^*fl/*−^ *EpoR-*Cre (+) × *Sec23a*^*fl/+*^ *Sec23b*^*fl/fl*^**						
**Genotypes**	***Sec23a*^*fl/+*^ *Sec23b*^*fl/−*^** ***EpoR*-Cre (+) #**	***Sec23a*^*fl/+*^ *Sec23b*^*fl/fl*^** ***EpoR*-Cre (+) #**	**Others**			***P* value^j^**
**Expected ratios**	12.5%	12.5%	75%			
Observed at E14.5% (*n* = 7)	57% (4)	0% (0)	43% (3)			0.0495
**K. *Sec23b*^*fl/−*^ *EpoR-*Cre (+) × *Sec23a*^*fl/−*^ *Sec23b*^*fl/+*^**						
**Genotypes**	***Sec23a*^*fl/+*^ *Sec23b*^*fl/fl*^** ***EpoR*-Cre (+) #**	***Sec23a*^*fl/+*^ *Sec23b*^*fl/−*^** ***EpoR*-Cre (+) #**	***Sec23a*^−/+^ *Sec23b*^*fl/fl*^** ***EpoR*-Cre (+) #**	***Sec23a*^*−/+*^ *Sec23b*^*fl/*−^** ***EpoR*-Cre (+) #**	**Others**	***P* value^k^**
**Expected ratios**	6.25%	6.25%	6.25%	6.25%	75%	
Observed at E14.5% (*n* = 6)	33% (2)	0% (0)	17% (1)	0% (0)	50% (3)	0.15
**L. *Sec23b*^*fl/−*^ × *Sec23a*^*fl/fl*^ *Sec23b*^*fl/+*^** ***EpoR-*Cre (+)**						
**Genotypes**	***Sec23a*^*fl/+*^ *Sec23b*^*fl/fl*^** ***EpoR*-Cre (+) #**	***Sec23a*^*fl/+*^ *Sec23b*^*fl/−*^** ***EpoR*-Cre (+) #**	**Others**			***P* value^l^**
**Expected ratios**	12.5%	12.5%	75%			
Observed at E14.5% (*n* = 6)	0% (0)	17% (1)	83% (5)			0.64
**M. *Sec23a*^−/*fl*^** ***EpoR-*Cre (+) × *Sec23a*^+/−^ *Sec23b*^*+/−*^**						
**Genotypes**	***Sec23a*^−/*fl*^ *Sec23b*^+/−^** ***EpoR*-Cre (+) #**	**Others**				***P* value^m^**
**Expected ratios**	**8.3%**	**91.7%**				
Observed at weaning % (*n* = 161)	7.5% (12)	92.5% (149)				0.70
**N. *Sec23a*^−/*fl*^ *EpoR-*Cre (+) × *Sec23a*^*fl/fl*^ *Sec23b*^*fl/fl*^**						
**Genotypes**	***Sec23a*^*fl/fl*^ *Sec23b*^*+/fl*^** ***EpoR*-Cre (+) #**	***Sec23a*^−/*fl*^ *Sec23b*^*+/fl*^** ***EpoR*-Cre (+) #**	**Others**			***P* value^n^**
**Expected ratios**	**25%**	**25%**	**50%**			
Observed at weaning % (*n* = 16)	18.8% (3)	25% (4)	56.2% (9)			0.62
**O. *Sec23a*^*fl/fl*^ *EpoR-*Cre (+) × *Sec23a*^+/−^ *Sec23b*^*+/−*^**						
**Genotypes**	***Sec23a*^−/*fl*^ *Sec23b*^+/−^** ***EpoR*-Cre (+) #**	**Others**				***P* value^o^**
**Expected ratios**	**12.5%**	**87.5%**				
Observed at weaning % (*n* = 12)	25% (3)	75% (9)				0.19

*Sec23b^−/−^ Sec23^b-a^* BAC^+^ mice exhibited normal growth and had an average adult weight indistinguishable from that of WT littermate controls (Fig. 1D). In contrast to the massive pancreatic degeneration observed in *Sec23b^−/−^* mice, pancreatic tissues harvested from *Sec23b^−/−^ Sec23^b-a^* BAC^+^ mice appeared normal histologically by hematoxylin and eosin (H&E) stain (Fig. 1E). Similarly, *Sec23b^−/−^ Sec23^b-a^* BAC^+^ pancreas tissues exhibited normal ER structure and intact acinar zymogen granules, in contrast to the dilated ER observed in *Sec23b^−/−^* pancreas tissues (Fig. 1F). Furthermore, a cohort of 12 *Sec23b^−/−^ Sec23^b-a^* BAC^+^ mice was followed for more than 1.5 years, demonstrating normal survival of these mice with no apparent abnormalities by routine necropsy. Collectively, these results demonstrate that the SEC23A protein functionally overlaps with SEC23B.

### Erythroid-specific SEC23A-deficient mice do not exhibit an erythroid defect

We have previously demonstrated that in contrast to humans, mice with erythroid-specific SEC23B deficiency do not exhibit anemia or a defect in erythroid maturation (*12*). Given the functional overlap between SEC23A and SEC23B, we reasoned that erythroid deficiency for SEC23A alone or in combination with SEC23B may result in an RBC phenotype in the mouse.

**Fig. 2.**
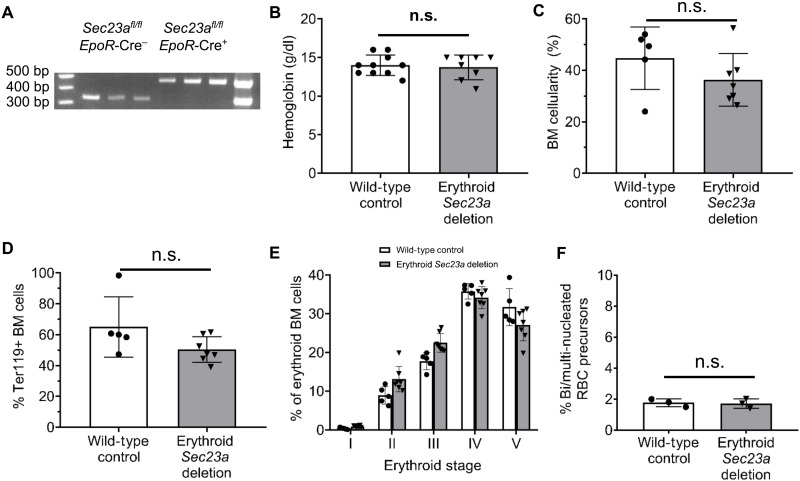
Erythroid-specific SEC23A deficiency does not result in a murine erythroid phenotype. (**A**) Erythroid cells isolated from *Sec23a^fl/fl^ EpoR*-Cre^+^ mice had no detectable *fl* allele by PCR. (**B**) Hemoglobin levels, (**C**) BM cellularity, (**D**) percentage of BM Ter119-positive cells, and (**E**) distribution of BM erythroid cells among five stages of erythroid differentiation are similar in mice with erythroid-specific *Sec23a* deletion (*EpoR*-Cre) and WT littermate controls. (**F**) The BM of mice with erythroid-specific *Sec23a* deletion had the same percentage of bi- or multinucleated erythroid precursors as WT littermate controls. Abbreviations: BM, bone marrow; RBC, red blood cell. Two-sided, unpaired *t* tests were used to compare means except in (E), where two-way analysis of variance (ANOVA) and Sidak’s multiple comparisons test were performed. *P* values not significant (n.s.) if not marked. Data represent means ± SD.

Germline SEC23A deficiency (*Sec23a^−/−^*) has been previously shown to result in embryonic lethality at E11.5 (*26*). Therefore, to define the role of SEC23A in erythropoiesis, we crossed the conditional *Sec23a* floxed allele (*Sec23a^fl^*), in which exon 3 is flanked by loxP sites, to a mouse expressing Cre recombinase in erythroid cells under the control of the *EpoR* promoter (*EpoR*-Cre). Mice with erythroid-specific *Sec23a* deletion (*Sec23a^−/fl^ EpoR*-Cre^+^ or *Sec23a^fl/fl^ EpoR*-Cre^+^ mice) were generated using various mating schemes as demonstrated in Table 1 (B and C); these mice were recovered at the expected Mendelian ratios at weaning (Table 1, B and C). Erythroid cells were sorted from *Sec23a^fl/fl^ EpoR*-Cre^+^ mice and demonstrated no detectable *fl* allele by polymerase chain reaction (PCR), consistent with complete (or near complete) excision of the *Sec23a^fl^* allele in the erythroid cells (Fig. 2A).

Mice with erythroid-specific SEC23A deficiency exhibited no anemia (Fig. 2B). BM cellularity (Fig. 2C), BM morphology (fig. S1), percent of BM erythroid cells (Fig. 2D), and the distribution of erythroid cells among the five sequential stages of maturation (Fig. 2E) were indistinguishable between mice with erythroid-specific SEC23A deficiency and WT littermate controls. In addition, the percentage of BM binucleated erythroid precursors was not increased in *Sec23a^fl/fl^ EpoR*-Cre^+^ mice (Fig. 2F). These data demonstrate that erythroid-specific SEC23A deficiency does not result in an erythroid defect in the mouse.

### Combined SEC23A/SEC23B deletion in mouse erythroid cells is lethal

**Fig. 3.**
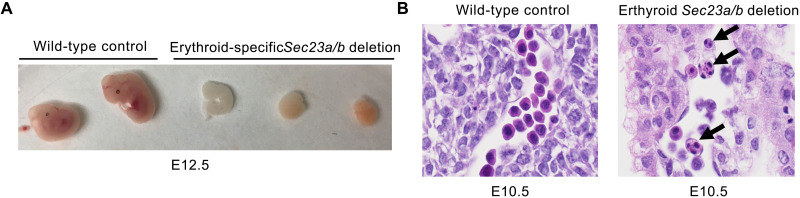
Mice with combined erythroid-specific SEC23A and SEC23B deficiency. (**A**) Mice with combined erythroid-specific (*EpoR*-Cre) *Sec23a*/*Sec23b* deletion die at mid-embryogenesis (E12.5) exhibiting a smaller size and pale appearance compared to their WT littermate controls and (**B**) dyserythropoiesis (black arrows) on histological evaluation (E10.5).

Multiple mating schemes outlined in Table 1 (D and E) demonstrated that mice with combined SEC23A/B deficiency in the erythroid lineage (*Sec23a^fl/fl^ Sec23b^fl/fl^ EpoR*-Cre^+^, *Sec23a^fl/fl^ Sec23b^−/fl^ EpoR*-Cre^+^, and *Sec23a^−/fl^ Sec23b^−/fl^ EpoR*-Cre^+^) are not viable at weaning. Mice with combined erythroid-specific SEC23A/B deficiency (Table 1F) die at mid-embryogenesis (E12.5), exhibiting reduced size and appearing pale compared to their WT littermate controls (Fig. 3A). Notably, mice with combined deletion of both *Sec23* paralogs in erythroid cells exhibit histologic evidence of dyserythropoiesis reminiscent of human CDAII (Fig. 3B). Together, these results are consistent with the requirement for a threshold level of total SEC23 (combined SEC23A/B) expression in the erythroid compartment.

### Deletion of three *Sec23* alleles in the mouse erythroid cells results in an erythroid defect

**Fig. 4.**
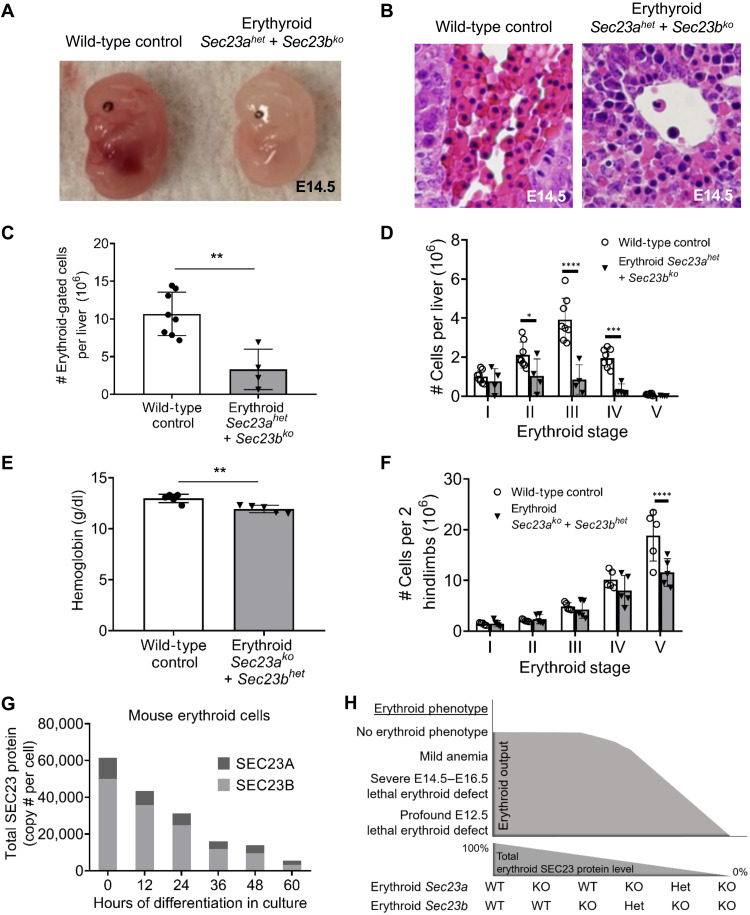
Erythroid phenotypes of mice with deletion of three of the four *Sec23* alleles using the *EpoR*-cre. (**A**) Mice with combined erythroid-specific *Sec23a* haploinsufficiency and *Sec23b* deletion (*Sec23a^het^* + *Sec23b^ko^*) die in late embryogenesis, with E14.5 embryos exhibiting pale appearance, (**B**) reduced fetal liver erythropoiesis by both H&E staining and (**C**) flow cytometry analysis, as well as (**D**) impaired erythroid maturation in the fetal liver as determined by flow cytometry (see fig. S2B for gating strategy); *n* = 6 for WT controls, *n* = 4 for erythroid *Sec23a^het^* + *Sec23b^ko^*. (**E**) Mice with combined erythroid-specific *Sec23a* deletion and *Sec23b* haploinsufficiency (*Sec23a^ko^* + *Sec23b^het^*) are viable and exhibit a ~1.0 g/dl reduction in hemoglobin level compared to WT littermate controls and (**F**) impaired terminal erythroid maturation in the BM as determined by flow cytometry (*n* = 5 for each group). (**G**) SEC23B is more abundant than SEC23A in murine erythroid cells. Data were obtained from Gautier *et al.* (*28*). The 0 hour of differentiation consisted primarily of erythroid colony-forming units and proerythroblasts, while hour 60 of differentiation consisted primarily of orthochromatic erythroblasts and reticulocytes. (**H**) The severity of the erythroid phenotype in the mouse is inversely correlated with the total SEC23 level in erythroid cells. Statistical tests: Two-sided, unpaired *t* tests were used in (C) and (E); two-way ANOVA with Sidak’s multiple comparisons were used in (D) and (F). *P* values: *P < 0.05, **P < 0.01, ***P < 0.001, ****P < 0.0001. *P* values not significant (n.s.) unless otherwise marked. Data represent means ± SD.

We next generated mice with combined *Sec23a* haploinsufficiency and biallelic *Sec23b* deletion in the erythroid compartment (erythroid-specific *Sec23a^het^ Sec23b^ko^* mice), using various mouse crosses as described in Table 1 (G to I). These latter mice were not viable at weaning (Table 1, G to I). Timed matings (Table 1, J to L) demonstrated that erythroid-specific *Sec23a^het^ Sec23b^ko^* mice die at ~E14.5 to 16.5, exhibiting pale appearance compared to WT littermate controls (Fig. 4A). Livers harvested from E14.5 erythroid-specific *Sec23a^het^ Sec23b^ko^* animals contained reduced numbers of erythroid cells compared to WT littermate control livers, as demonstrated by histologic evaluation (Fig. 4B) and flow cytometry (Fig. 4C). *Sec23a^het^ Sec23b^ko^* erythroid cells exhibited impaired maturation by flow cytometry, with reduced stage II, III, and IV erythroblasts, reminiscent of severe human CDAII (Fig. 4D and fig. S2A) (*27*). *Sec23a^het^ Sec23b^ko^* erythroid cells also exhibited increased binucleated erythroblasts on cytospin and histologic evaluation of fetal liver cells, a characteristic CDAII feature (fig. S3).

We subsequently generated mice with combined biallelic *Sec23a* deletion and *Sec23b* haploinsufficiency in the erythroid compartment (erythroid-specific *Sec23a^ko^ Sec23b^het^*) using several mating schemes as described in Table 1 (M to O). These mice were not underrepresented at weaning and had normal life spans. However, compared to WT littermate controls, erythroid-specific *Sec23a^ko^ Sec23b^het^* mice exhibited a mild reduction in hemoglobin levels (*P* < 0.01) (Fig. 4E) and a block in late erythroid maturation in the BM by flow cytometry (Fig. 4F and fig. S2B).

### Expression of the SEC23 paralogs in mouse erythroid cells

We next evaluated the relative expression of SEC23B and SEC23A in WT murine erythroid cells using recently published proteomic analysis of murine erythroid cells at various stages of terminal maturation (*28*). Notably, the SEC23B protein level was found to be higher than SEC23A at all stages of erythroid differentiation (Fig. 4G). On the basis of the differential expression of the SEC23 paralogs in mouse erythroid cells, as well as the data describing the erythroid phenotypes observed in mice with deletion of various combinations of the *Sec23* alleles, we conclude that (i) SEC23A functionally overlaps with SEC23B in murine erythroid cells, and (ii) the severity of the murine erythroid defect inversely correlates with the total SEC23 protein (SEC23A + SEC23B) level in erythroid cells (Fig. 4H).

### *SEC23B*-deleted human erythroid HUDEP-2 cell lines exhibit features of CDAII

We have shown that SEC23A compensates for SEC23B deficiency in murine erythroid cells. To determine whether SEC23A also functionally compensates for SEC23B deficiency in human erythroid cells, we first generated clonal human erythroleukemia K562 cell lines that are deficient in SEC23B using CRISPR-Cas9 genome editing. We confirmed biallelic frameshift insertions/deletions (indels) in exon 2 of *SEC23B* by PCR and Sanger sequencing and subsequently verified deficiency of the SEC23B protein by immunoblotting (fig. S4A). Notably, the *SEC23B*-targeting single-guide RNA (sgRNA) used in this manuscript did not target *SEC23A* (fig. S4B). Compared to control WT K562 clonal cell lines, which were generated using a nontargeting sgRNA, SEC23B-deficient K562 cells exhibited no increased percentage of binucleated cells either before or following differentiation into erythroid cells with hemin (fig. S4, C and D). In addition, we generated SEC23A-deficient K562 cell lines (fig. S4B) and similarly demonstrated no increased percentage of binucleated cells at baseline or following hemin-mediated erythroid differentiation (fig. S4, C and D). K562 cells with combined deficiency for both SEC23A and SEC23B could not be generated, suggesting that combined *SEC23A*/*SEC23B* deletion is not tolerated in these cells.

**Fig. 5.**
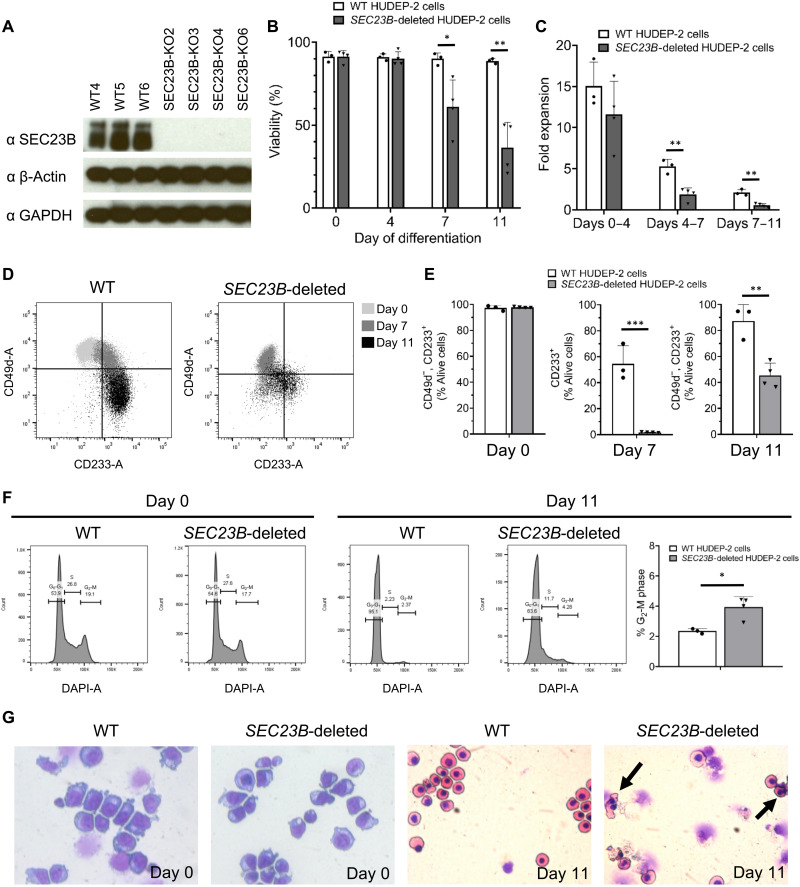
SEC23B-deficient HUDEP-2 cells exhibit an erythroid defect. (**A**) *SEC23B*-deleted HUDEP-2 cells exhibit absence of detectable SEC23B protein by immunoblotting. (**B** and **C**) During the initial days of differentiation, *SEC23B-*deleted and WT HUDEP-2 cells exhibit comparable viability and fold expansion; however, during subsequent days of differentiation, SEC23B-deficient cells exhibit reduced viability and impaired expansion compared to WT cells. (**D** and **E**) SEC23B-deficient HUDEP-2 cells exhibit impaired up-regulation of CD233 during differentiation. (**F**) SEC23B-deficient HUDEP-2 cells exhibit normal distribution of cells among the various phases of the cycle at day 0 of differentiation but increased percentage of cells in the G_2_-M phase at day 11 of differentiation. (**G**) SEC23B-deficient HUDEP-2 cells exhibit normal erythroid morphology during the expansion phase but increased erythroid binuclearity at day 11 of differentiation (arrows), as demonstrated by cytospin evaluation. Statistical tests: Two-sided, unpaired *t* tests were used to compare means. *P* values: *P < 0.05, **P < 0.01, ***P < 0.001. *P* values not significant (n.s.) unless otherwise marked. Data represent means ± SD.

Since we did not observe a clear defect in SEC23B-deficient K562 cells, and since the levels of SEC23A and SEC23B proteins are comparable in K562 cells (fig. S6A) (*29*), while human erythroid cells express substantially higher levels of SEC23B compared to SEC23A (*30*), we next deleted *SEC23B* in the HUDEP-2 cell line using CRISPR-Cas9–mediated genome editing. The HUDEP-2 immortalized cell line can be induced to differentiate into mature erythroid cells and is a good model of human terminal erythroid maturation (*31*). We generated four independent HUDEP-2 clonal lines with confirmed biallelic frameshift mutations in *SEC23B* (fig. S5, A and B), resulting in a stop codon in exon 2 of *SEC23B* and undetectable SEC23B protein by immunoblotting (Fig. 5A). SEC23B-deficient HUDEP-2 cells survive, grow, and expand normally (fig. S5C and Fig. 5, B and C) compared to WT HUDEP-2 cells when maintained as undifferentiated early erythroid cells in expansion media. However, upon culturing the cells in differentiation media, differentiated SEC23B-deficient HUDEP-2 cells exhibited reduced cell viability (Fig. 5B), reduced expansion (Fig. 5C), and impaired expression of surface CD233 (Fig. 5, D and E) compared to similarly differentiated WT HUDEP-2 cells. In addition, *SEC23B*-deleted HUDEP-2 cells exhibited an increased percentage of binucleated cells upon differentiation (Fig. 5, F and G, and fig. S5D), a characteristic feature of CDAII. The late erythroid differentiation defects observed in SEC23B-deficient HUDEP-2 cells is consistent with the significant reduction of SEC23A levels throughout erythroid differentiation, as demonstrated by quantitative mass spectrometry data (fig. S6B) (*30*), flow cytometry (fig. S6C), and Western blot (fig. S6, D and E).

### SEC23A and SEC23B overlap in function in human erythroid cells

**Fig. 6.**
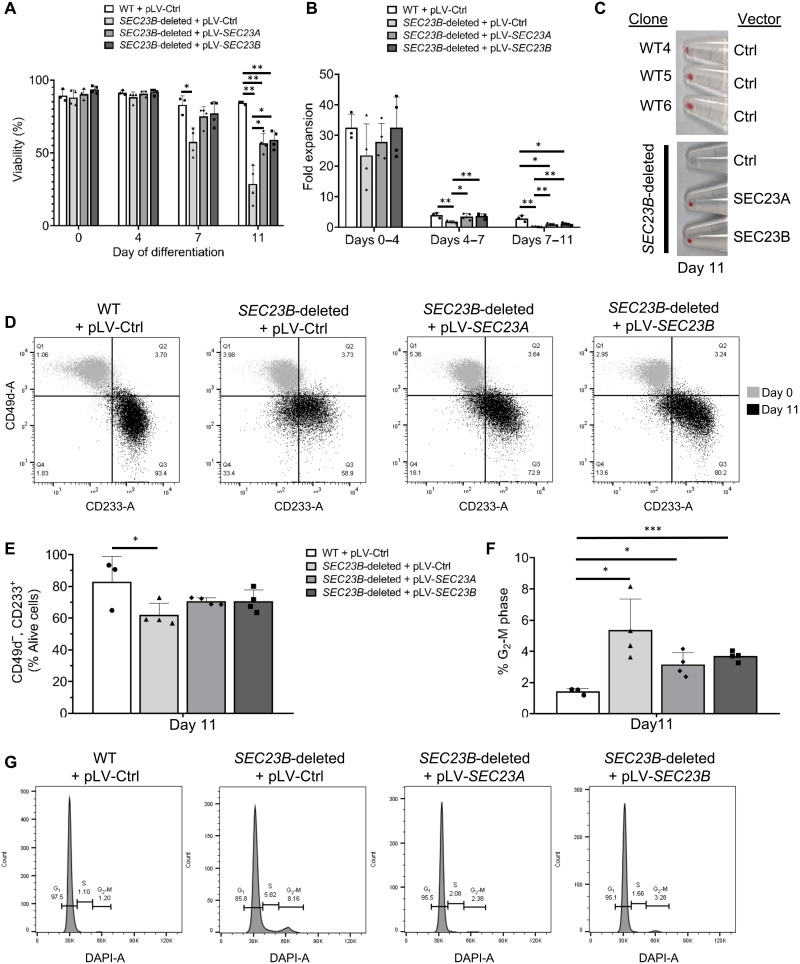
SEC23A overlaps in function with SEC23B in human erythroid cells. SEC23B-deficient HUDEP-2 cells were transduced with lentivirus that express SEC23A-HA, SEC23B-HA, or neither as negative control. Upon differentiation, compared to WT HUDEP-2 cells, SEC23B-deficient HUDEP-2 cells exhibit (**A**) impaired viability, (**B**) reduced expansion, (**C**) impaired hemoglobinization, (**D** and **E**) impaired terminal erythroid maturation (assessed by flow cytometry), and (**F** and **G**) increased percentage of differentiated erythroid cells with binuclearity by flow cytometry. All the defects observed in SEC23B-deficient HUDEP-2 cells are rescued to similar extents by expression of either SEC23B or SEC23A. Statistical tests: Two-sided, unpaired *t* tests were used to compare means. *P* values: *P < 0.05, **P < 0.01, ***P < 0.001. *P* values not significant (n.s.) unless otherwise marked. Data represent means ± SD.

To determine whether SEC23A can functionally substitute for SEC23B in human erythroid cells, *SEC23B*-deleted HUDEP-2 cells were transduced with lentivirus that expressed either hemagglutinin (HA)–tagged SEC23A (SEC23A-HA) or HA-tagged SEC23B (SEC23B-HA), or with an empty vector as control. As expected, relatively similar levels of SEC23A and SEC23B proteins were achieved by lentiviral expression of SEC23A-HA or SEC23B-HA, as demonstrated by immunoblotting for the HA tag (fig. S7A). Notably, the reduced cell viability (Fig. 6A), impaired expansion (Fig. 6B), hemoglobinization defect (Fig. 6C), differentiation defect (Fig. 6, D and E), and increased binuclearity (Fig. 6, F and G) observed in SEC23B-deficient HUDEP-2 cells were largely rescued by expression of *SEC23B* from the SEC23B-HA lentiviral construct, ruling out an off-target effect of the sgRNA used to generate the *SEC23B*-deleted HUDEP-2 cells. The level of SEC23B expression obtained with the SEC23B-HA lentiviral construct was ~5% that of the endogenous SEC23B level (fig. S7B), likely explaining the incomplete rescue achieved with this vector. The differentiation defects resulting from SEC23B deficiency were also equally rescued by expression of *SEC23A* cDNA from the same lentiviral construct (Fig. 6, A to G). These data demonstrate that SEC23A overlaps in function with SEC23B in human erythroid cells.

### CRISPRa-mediated SEC23A activation rescues the SEC23B-deficient erythroid defects

**Fig. 7.**
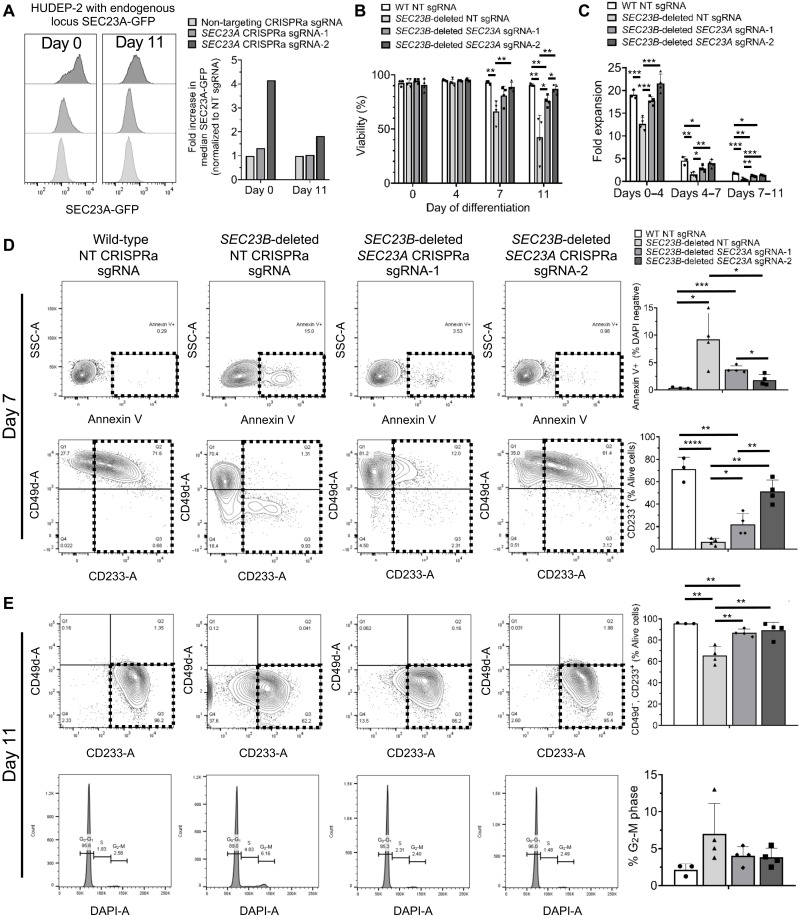
Erythroid defects observed in SEC23B-deficient HUDEP-2 cells are rescued in a dose-dependent manner by increased expression of SEC23A using CRISPR-activation (CRISPRa). (**A**) Two independent *SEC23A*-targeting CRISPRa gRNAs were individually transduced into a HUDEP-2 reporter cell line expressing GFP-tagged SEC23A from the endogenous *SEC23A* locus. GFP fluorescence was quantified by flow cytometry at days 0 and 11 of differentiation and compared to cells transduced with a NT control gRNA. (**B**) Viability and (**C**) fold expansion of SEC23B-deficient HUDEP-2 cells (*n* = 4 independent clonal lines) are rescued by increased expression of SEC23A, in a dose-dependent manner. (**D** and **E**) SEC23B-deficient HUDEP-2 cells were evaluated at days 7 and 11 of differentiation and found to exhibit increased annexin V staining, impaired up-regulation of surface CD233, and increased % of cells at the G_2_-M phase of the cell cycle (consistent with increased binucleated cells). All these defects were ameliorated by activation of SEC23A, using either sgRNA1 or sgRNA2, mostly in a dose-dependent manner. Statistical tests: Two-sided, unpaired *t* tests were used to compare means. *P* values: *P < 0.05, **P < 0.01, ***P < 0.001, ****P < 0.001. *P* values not significant (n.s.) unless otherwise marked. Data represent means ± SD.

We next assessed whether increased expression of SEC23A from its endogenous locus using CRISPRa rescues the erythroid defects observed in SEC23B-deficient HUDEP-2 cells. To address this, we first generated a HUDEP-2 reporter cell line that expresses green fluorescent protein (GFP)–tagged SEC23A from the endogenous *SEC23A* locus (fig. S8A). Similar to CD34-differentiated erythroid cells (fig. S6B), SEC23A expression in this reporter cell line decreases with erythroid differentiation (fig. S6C). Using this reporter cell line, we show that two independent *SEC23A*-targeting sgRNAs, CRISPRa *SEC23A* sgRNA-1 and sgRNA-2, respectively increased SEC23A level by ~30 and ~300% in undifferentiated HUDEP-2 cells and by ~5 and ~80% at day 11 of differentiation, as determined by flow cytometry (Fig. 7A). Increasing the expression of SEC23A with either of the two sgRNAs did not result in any detectable differentiation defect in the reporter cell line or in three independent WT HUDEP-2 clonal cell lines (fig. S8, B to E). Subseqently, we tested the ability of both SEC23A-targeting sgRNAs to rescue the defects observed in SEC23B-deficient HUDEP-2. The decreased viability (Fig. 7B), reduced fold expansion (Fig. 7C), increased annexin V staining (Fig. 7D), impaired surface CD233 up-regulation (Fig. 7, D and E), and binuclearity (Fig. 7E) observed in SEC23B-deficient HUDEP-2 cells were rescued by either of the two SEC23A-targeting sgRNAs in a dose-dependent manner. In addition, SEC23B-deficient HUDEP-2 cells exhibit a narrower size of the RBC membrane protein band 3 by Western blot, a specific characteristic feature of CDAII, and this phenotype is rescued by increased expression of SEC23A (fig. 9, A to B). These data demonstrate that increasing SEC23A expression from the endogenous locus rescues the erythroid defects exhibited by SEC23B-deficient erythroid cells.

## DISCUSSION

Gene duplication is a common evolutionary event. Most frequently, one of the copies of the duplicated gene acquires loss-of-function mutations and is subsequently lost over evolutionary time. Less commonly, both copies (paralogs) of the duplicated gene become fixed in the genome, either because one paralog acquires a new function (neofunctionalization) or because both paralogs split the function of the ancestral gene (subfunctionalization). Regarding the *SEC23* duplication, it is estimated to have occurred ~615 million years ago (*17*). However, notably, the SEC23A and SEC23B proteins share a high degree of amino acid sequence identity (~85%) (*16*), yet previous studies suggested unique functions for SEC23A and SEC23B (*18*–*25*). Here, we generated mice expressing the complete SEC23A protein under the control of *Sec23b* regulatory elements and demonstrated that full-length SEC23A completely rescues the lethality and pancreatic phenotype of SEC23B-deficient mice. These data conclusively demonstrate a functional overlap between SEC23A and SEC23B in vivo. These results are also consistent with the previously demonstrated identical interactomes for SEC23A and SEC23B in human embryonic kidney (HEK) 293T cells and complementation of yeast Sec23 by both SEC23 paralogs (*17*). Although the interactomes of SEC23A and SEC23B are identical in HEK293 cells, future work that compares the SEC23A and SEC23B interactomes in human erythroid cells would be of interest.

We previously showed that in contrast to humans, mice with hematopoietic SEC23B deficiency do not exhibit anemia or other CDAII characteristics (*12*). Although SEC23A and SEC23B appear to be functionally interchangeable in vivo, the divergent phenotypes of SEC23B deficiency between mice and humans remained puzzling. The expression profiles of SEC23A and SEC23B were examined in various human tissues/cell types (*17*, *28*, *30*, *32*–*35*). SEC23B is the predominantly expressed paralog in human erythroid cells, with very little SEC23A expressed; this explains the inability of SEC23A to compensate for loss of SEC23B in CDAII erythroid cells, while other tissues appear to express adequate levels of SEC23A, which likely explains the absence of nonerythroid phenotypes in patients with CDAII. In contrast, murine erythroblasts express a higher level of SEC23A compared to human erythroblasts, consistent with lack of a CDAII-like phenotype in mice with hematopoietic SEC23B deficiency. Together with the functional overlap between the two SEC23 paralogs, these results suggest that the disparate phenotypes of SEC23B deficiency in mouse and humans may be due to an evolutionary change in the expression pattern of the two *SEC23* paralogs.

Therefore, in light of the data summarized above, we reasoned that combined deficiency for SEC23A and SEC23B may result in an erythropoiesis defect in mice. Here, we generated mice with erythroid-specific deletion of various combinations of the *Sec23a* and *Sec23b* alleles. While deletion of either *Sec23a* or *Sec23b* alone did not result in an erythroid defect, deletion of all four *Sec23* alleles resulted in a profound, embryonic lethal, erythroid defect, and deletion of three of the four *Sec23* alleles resulted in an intermediate phenotype. Although we cannot definitively exclude the possibility of subtle erythroid-specific differences in functions or cargo selectivity between the two SEC23 paralogs, these data are consistent with a functional overlap between SEC23A and SEC23B in the murine erythroid compartment. In addition, these results demonstrated an inverse correlation between the total level of SEC23 expression in erythroid cells and the severity of the erythroid phenotype, consistent with a threshold of total SEC23 level that is required for erythroid maturation. While the impact of deleting various combinations of *Sec23* genes in erythroid cells has been well characterized here, the impact of deleting various combinations of the *Sec23* genes in nonerythroid hematopoietic cells remains unknown and is of future interest.

As described above, we now report a murine model of human CDAII, which will be of critical importance to test new CDAII therapies in the future (*36*). Notably, this report has implications not only for CDAII but also for other human genetic diseases that result from loss-of-function mutations in one of two (or several) paralogous genes, for which deletion of the orthologous genes in mice result in no phenotypes (*37*–*40*). Our findings suggest that a strategy similar to the one implemented here could be applied in such disorders to generate mice that recapitulate the human phenotypes (*41*).

Down-regulation of *SEC23B* in K562 cells [using a *SEC23B*-targeting short hairpin RNA (shRNA)] was previously reported to result in increased binucleated cells (*4*). In contrast, we did not observe a similar increase in binucleated cells upon deletion of *SEC23B* in K562 cells using CRISPR-Cas9–mediated genome editing. The discrepancy between our results and the published results may be explained by differences in the methods used to generate SEC23B-depleted cells. One possible explanation is that the *SEC23B*-targeting shRNA used in the previously published report also down-regulated SEC23A, resulting in reduced total SEC23 level (*36*). In contrast, we showed that the *SEC23B*-targeting sgRNAs we used here did not target *SEC23A*. Another possible explanation is that the levels of SEC23A and SEC23B proteins are comparable in K562 cells (*29*), while human erythroid cells express significantly higher levels of SEC23B compared to SEC23A (*30*). HUDEP-2 is an immortalized human erythroid cell line that has been used to investigate several questions in erythropoiesis (*31*, *42*–*47*) and globin switching (*48*–*60*), appears to mimic human erythroid maturation more faithfully than K562 cells, and expresses SEC23A/B in a comparable pattern to human erythroid cells. Here, we generated SEC23B-deficient HUDEP-2 cell lines; these cell lines exhibited erythroid maturation defects and an increased percentage of binucleated erythroblasts, both of which are features of CDAII. We showed that the differentiation defects resulting from SEC23B deficiency are equally rescued by increased expression of SEC23A or SEC23B, demonstrating that the functional overlap between the SEC23 paralogs is conserved in human erythropoiesis. The findings here raise the possibility that the severity of the CDAII phenotype may depend, at least in part, on the level of expression of SEC23A in the erythroid compartment. CDAII patients with higher expression of erythroid SEC23A appear to exhibit a milder phenotype than those expressing lower SEC23A levels (*61*).

Collectively, the findings here suggest that strategies aimed at increasing the expression of SEC23A in erythroid cells may be therapeutically effective in CDAII. While the global safety of increasing SEC23A expression requires more comprehensive study in vitro and in vivo, overexpression of SEC23A in WT HUDEP-2 cells here did not result in any detectable defect. This is consistent with previous work in which overexpression of SEC23A in zebrafish had no identified detrimental effect (*17*).

Whether SEC23A expression can be increased pharmacologically remains unknown and is an important area of future investigation. The transcription factor CREB3L2/BBF2H7 has been previously shown to increase SEC23A expression (*62*–*64*); however, CREB3L2/BBF2H7 alters the expression of a large number of genes (*63*), likely limiting its therapeutic potential. Alternative approaches to increase SEC23A expression include CRISPR-based approaches (such as CRISPR activation strategies). We demonstrate here the feasibility and efficacy of this approach by showing that increased expression of SEC23A using CRISPRa rescues the SEC23B-deficient erythroid defect, suggesting a novel strategy to treat CDAII. We have further shown via studies in HUDEP-2 cells and in mice with erythroid-specific deletion of a combination of *Sec23a/b* alleles that the CDAII defect is intrinsic to the erythroid cell. Therefore, genetic strategies aimed at increasing SEC23A levels therapeutically for CDAII may be performed exclusively in erythroid cells, limiting the possibility of complications in other cells, as recently shown for other erythroid disorders such as sickle cell disease (*65*).

The therapeutic implications of this work extend beyond CDAII to other disorders resulting from mutations in one of two or more paralogous genes, for which increasing the expression of a nonaffected functionally overlapping paralog may be of clinical benefit. For instance, increasing the expression of *SMN2* may have clinical utility in patients with spinal muscle atrophy owing to *SMN1* mutations (*66*–*71*), and increased expression of *PAX2* or *PAX8* may be of interest in patients with predisposition to acute lymphoblastic leukemia due to *PAX5* mutations (*72*). Therefore, findings here lay the foundation for future work that aims to examine a novel therapeutic strategy in various disorders.

## METHODS

### *Sec23a* and *Sec23b* mutant alleles

A *Sec23b* floxed allele (*Sec23b^fl^*) in which exons 5 and 6 are flanked by LoxP sites and a *Sec23b* null allele (*Sec23b^−^*) with excision of *Sec23b* exons 5 and 6 were generated as previously described (*9*, *10*, *12*). In addition, a *Sec23a* floxed allele (*Sec23a^fl^*) in which exon 3 is flanked by *loxP* sites and a *Sec23a* null allele (*Sec23a^−^*) with deletion of exon 3 were also generated as previously described (*10*). All *Sec23* alleles were backcrossed to C57BL/6J mice for >10 generations and subsequently maintained on a pure C57BL/6J background.

### Recombineering the *Sec23a* cDNA into the *Sec23b* locus

A ~170-kb mouse BAC containing *Sec23b* [RP24-371A4 (RP24)] was purchased from the BACPAC Resources Center. We have previously shown that the key regulatory sequences required for *Sec23b* expression reside in a 127-kb region that is contained within the 170-kb RP24 BAC (*10*). A donor vector that contains the following sequences from the 5′ to 3′ direction was assembled: 5′ homology sequence with RP24, *Sec23a* cDNA synthesized de novo (by GenScript) without the stop codon, a sequence encoding three glycines followed by an HA tag and three stop codons, a bovine growth hormone polyadenylation transcriptional termination sequence, a kanamycin resistance cassette with its *PGK* promoter flanked by two FRT sites, and a 3′ homology sequence with RP24 (Fig. 1A). This vector allows insertion of the *Sec23a* cDNA at the translational start of *Sec23b* by homologous recombination without disrupting/removing any of the BAC sequence. The RP24 BAC clone and the donor vector were combined in recombineering competent bacteria as previously described (*73*). Sequence integrity of the inserted cDNA and the insert junctions was confirmed by Sanger sequencing. Successful recombinants were selected with kanamycin, and subsequently, the kanamycin resistance cassette was removed by expression of FLP recombinase (*73*). The recombineered BAC (*Sec23^b-a^* BAC) was purified using the Macherey-Nagel NucleoBond Xtra Maxi EF kit (#740424.10; Macherey-Nagel).

### Generation of mice that express *Sec23a* under the control of *Sec23b* regulatory elements

The recombineered *Sec23^b-a^* BAC that expresses *Sec23a* under the control of the *Sec23b* regulatory elements was microinjected into the pronucleus of zygotes generated from the in vitro fertilization of C57BL/6J oocytes with sperm from *Sec23b^+/−^* male mice. Zygotes were subsequently implanted in pseudopregnant foster mothers. Pups were genotyped at 2 weeks of age, and germline transmission of the BAC transgene was confirmed in one mouse line by genotyping.

### *Sec23a* and combined *Sec23a*/*Sec23b* deletion in the erythroid compartment

To generate mice with erythroid-specific SEC23A deficiency, the *Sec23a^fl^* and the *Sec23a^−^* alleles were crossed to mice expressing Cre recombinase under the control of the erythropoietin receptor promoter (*EpoR*-Cre mice) [a gift from U. Klingmüller (*74*)] (Table 1, B and C). The *EpoR*-Cre mice were backcrossed on a C57BL/6J genetic background for >10 generations.

Mice with combined *Sec23a*/*Sec23b* deletion in the erythroid compartment were generated as described in Table 1 (D to F). In addition, mice with biallelic deletion of one of the *Sec23* paralogs and haploinsufficiency for the other paralog in the erythroid compartment were also generated, as described in Table 1 (G to O). All experiments using mice were approved by and performed in accordance with the regulations of the University Committee on Use and Care of Animals.

### Mouse genotyping

Genomic DNA was isolated from mouse tail biopsies using DirectPCR Lysis Reagent (Viagen BioTech #102-T) per the manufacturer’s instructions. PCR genotyping for *Sec23b^fl^*, *Sec23b^−^*, *Sec23a^fl^*, *Sec23a^−^*, and *Epor*-Cre was performed as previously described (*10*, *12*). Presence of the *Sec23^b-a^* BAC transgene was determined by PCR using primer pair BAC-F2 (CGGAAGTCAGGATACCAGGA) and BAC-R2 (TGACTCCCATAAGACTTGGACA), with one primer located in the *Sec23a* cDNA and one primer located in the RP24 BAC; therefore, this primer pair should not yield a detectable PCR product from the WT *Sec23a* or the WT *Sec23b* alleles. To distinguish between the *Sec23b^+/−^ Sec23^b-a^* BAC^+^ and the *Sec23b^−/−^ Sec23^b-a^* BAC^+^ genotypes, reverse transcription (RT)–PCR was performed on mRNA isolated from mouse tail biopsies using a forward primer (BAC-F3: CTTTAAAAGAACGCCCAGACTTAC) and a reverse primer (BAC-R3: CTCCCCGAGAAGATCTGTGA) located in *Sec23b* exons 2 and 7, respectively. To perform the latter analysis, mRNA was extracted from mouse tail biopsies using RNeasy Mini Kit (Qiagen) and RT was performed using the SuperScript First-Strand Synthesis System for RT-PCR (Invitrogen) with random hexamers per the manufacturers’ instructions.

### Complete blood counts

Blood was collected from the retro-orbital venous sinuses of anesthetized mice with anticoagulant-coated capillary tubes as previously described (*10*) and analyzed on the Advia120 whole-blood analyzer (Siemens) or Hemavet HV950 (Drew Scientific).

### Histology

Tissues were collected at necropsy, fixed in aqueous buffered zinc formalin (Z-fix, Anatech), processed, embedded in paraffin, sectioned at 4 μm, and stained with H&E. Mouse pancreas tissues were prepared and examined by a transmission electron microscope (JEOL 1400) as previously described (*11*). For cytospins, approximately 50,000 cells were spun onto a glass slide at 500 rpm for 3 min (Shandon Cytospin 2). After drying, the slides were stained with Hema 3 Stat Pack (Fisher Scientific 122-122911). To determine the number of bi- or multinucleated HUDEP-2 cells at day 11 of differentiation, 500 nucleated cells per slide were counted by an observer blinded to the genotype of the cells.

### Cell fixing, staining, and sorting

Blood was collected as described above. BM was flushed from mice femurs and tibias as previously described, using Dulbecco’s modified Eagle’s medium (Gibco) supplemented with 4% fetal bovine serum (Peak Serum cat. #PS-FB2). Mouse cells were stained with combinations of the following antibodies: anti-Ter119 (BioLegend #116220), anti-CD11b (BioLegend #101226), anti-CD44 (BioLegend #103012), anti-CD71 (BioLegend #113818 and #113815), anti-GR1 (BioLegend #103224), anti-B220 (BioLegend #103224), and anti-Ki-67 (BioLegend #652404). HUDEP-2 cells were stained with anti-CD49d (BioLegend #304314) and either anti-CD233 (IBGRL/NHS Blood and Transplant #9438B1) followed by a fluorophore-conjugated streptavidin antibody (BioLegend #405208) or anti-CD233 (IBGRL/NHS Blood and Transplant #9439F1) (*75*). DAPI (4′,6-diamidino-2-phenylindole; Sigma-Aldrich #D8417) was used to identify dead cells. Annexin-V (BioLegend #640906) staining was performed per the manufacturer’s instructions.

For cell cycle analysis, cells were stained first with a Live/Dead stain (Invitrogen #L34973 or #L34976), followed by surface antibody stains. Subsequently, cells underwent fixation and permeabilization using the BD Fixation/Permeabilization Solution Kit (catalog #554714) per the manufacturer’s instructions, followed by application of RNase (Sigma-Aldrich #10109169001) and DNA stain with DAPI (Sigma-Aldrich #D8417). Flow cytometry was performed on an LSRFortessa (BD Biosciences), SYNERGY (iCyt), MA-800 (Sony), or FACSAria III cell sorter (BD Biosciences). Gating for erythroid cells in the murine BM was based on previously validated strategies (*76*, *77*). FCS files were analyzed with FlowJo software (BD Life Sciences).

### Western blotting

Cells or homogenized tissues were incubated in radioimmunoprecipitation assay lysis buffer (Thermo Fisher Scientific #89900) supplemented with a protease inhibitor [cOmplete, EDTA-free (Roche #11873580001)] for 45 to 60 min on an end-over-end rotator at 4°C. Following centrifugation at 20,000*g* for 30 min, supernatants (lysates) were collected and either used immediately or stored at −80°C until use. Lysates were mixed with 4× Laemmli buffer (Bio-Rad #161-0747) supplemented with 2-Mercaptoethanol (Thermo Fisher Scientific #BP176-100), boiled at 95°C for 5 min, and loaded on a 4 to 12% NuPAGE bis-tris gel (Invitrogen #NP0323). SDS gel electrophoresis was performed using NuPAGE MOPS SDS running buffer (Invitrogen #NP0001). Separated proteins were transferred to a nitrocellulose membrane (Bio-Rad #162-0115) using the tris-glycine transfer buffer (Bio-Rad #161-0734). Membranes were blocked in 5% milk (w/v) in tris-buffered saline with 0.1% Tween (TBST) for 1 hour and then probed with primary antibody overnight. Membranes were washed three times in TBST, probed with secondary antibody, and washed again three times in TBST. For x-ray development, membranes were incubated with SuperSignal West Pico PLUS Luminol/Enhancer Solution (Thermo Fisher Scientific #1863096) and SuperSignal West Pico PLUS Stable Peroxide Solution (Thermo Fisher Scientific #1863097) and exposed to Amersham Hyperfilm enhanced chemiluminescence (General Electric #28906839). Rabbit anti-mouse SEC23A and rabbit anti-mouse SEC23B were generated as previously described (*12*). Rabbit polyclonal anti-SEC23A (Invitrogen PA5-28984), rabbit polyclonal anti-SEC23B (NovusBio NBP2-20279), mouse anti-HA (Abcam 18181), and mouse anti-band3 (Santa Cruz Biotechnology 133190) were used for HUDEP-2 cells. Anti–glyceraldehyde-3-phosphate dehydrogenase (EMD Millipore, MAB374, 6C5), anti–β-actin (Sigma-Aldrich, A5316, clone AC-74), and anti–α-tubulin (Cell Signaling Technology 38735) were used as loading controls.

### Generation of SEC23A- and SEC23B-deficient K562 cells

K562 clonal cell lines that are deficient for either SEC23B or SEC23A were generated using an sgRNA-targeting *SEC23B* exon 2 (GGAACGTGTGGCCTTCCAGC) or an sgRNA-targeting *SEC23A* exon 2 (TCAAGTCGACTGGAAGCTAC), respectively, as previously described (*78*). Briefly, sgRNAs were cloned into pSpCas9(BB)-2A-GFP (PX458) (a gift from F. Zhang; Addgene plasmid #48138) and nucleofected in K562 cells using Lonza 4D-Nucleofector Core Unit (catalog #AAF-1002B; program DZ-100). Single cells were sorted into each well of a 96-well plate, and frameshift insertions or deletions were confirmed by PCR across the expected Cas9 cut site, TOPO cloning (using the Zero Blunt TOPO PCR Cloning Kit, Life Technologies), and Sanger sequencing. Primer pairs SEC23B F1/SEC23B R1 and SEC23A F1/SEC23A R1 were used to determine *SEC23B* and *SEC23A* indels, respectively (SEC23B F1: ACACTGTGTTACATGACCCATCTTC; SEC23B R1: TGGAATTATCAGGCCAGGGAGA; SEC23A F1: ATATTGCTGAATGAGGGGAGAACA; SEC23A R1: AGGATTCAAAACTGCACGGCAA). Clonal K562 cell lines without *SEC23B* or *SEC23A* mutations were used as controls. K562 cells were differentiated into erythroid cells with hemin, as previously described (*79*). Erythroid cells at various stages of maturation (*80*) were sorted, and cytopsins were prepared and evaluated by an investigator blinded to the cell genotype.

### Generation of SEC23B-deficient HUDEP-2 cells

HUDEP-2 cells were a gift from Y. Nakamura (*31*). SEC23B-deficient HUDEP-2 clonal cells lines were generated and validated for their deletion for *SEC23B*, as described above. Frameshift mutations in exon 2 of *SEC23B* were confirmed, also as described above. WT HUDEP-2 clonal cell lines, generated using an empty PX458 vector, were used as controls.

### Generation of HUDEP-2 cells expressing GFP-tagged SEC23A from the endogenous *SEC23A* locus

HUDEP-2 cells were coelectroporated (Lonza 4D nucleofector) with a PX459 plasmid expressing an sgRNA (ATTAGCACTTCAAGCAGCAC) targeting the SEC23A locus immediately upstream of the stop codon and with a donor template allowing insertion of the GFP coding sequence by homology-directed repair. The donor template was assembled as previously described (*81*) in a pUC19 vector (pUC19 was a gift from J. Messing, Addgene plasmid #50005) (*82*) using the NEBuilder HiFi DNA assembly cloning kit [New England Biolabs (NEB)] with the following consecutive sequences: an ~800-base pair (bp) homology arm upstream of the sgRNA cut site, a sequence encoding a linker protein (GGAPAPAPAPAPAPAPAPG), the GFP-coding sequence followed by a stop codon, and an ~800-bp homology arm downstream of the sgRNA cut site (fig. S5A). Single cells were sorted into each well of a 96-well plate. Correct biallelic insertion of GFP at the *SEC23A* locus was confirmed by PCR using a primer pair flanking the homology sequences (forward primer GATAGGTCCTGTTATTATCCTTGTTTTAC and reverse primer AGCATCACATGATATAATTAAGCAAACAT), followed by Sanger sequencing.

### Cell culture

HUDEP-2 cells were cultured as previously described (*83*). Briefly, cells were maintained in expansion media consisting of StemSpan SFEM (STEMCELL Technologies, catalog #09650) supplemented with human recombinant stem cell factor (rhSCF, 50 ng/ml) (R&D, catalog #255-SC-01M), 1 μM dexamethasone (Cayman, catalog #11015), doxycycline (2 μg/ml; Sigma-Aldrich, catalog #D9891), Epogen (3 U/ml; Amgen), and penicillin-streptomycin (100 U/ml; Gibco, catalog #15140-122). Cells were passaged every 48 to 72 hours to maintain their density at 50,000 to 800,000 cells/ml. To induce erythroid differentiation, HUDEP-2 cells were switched to differentiation media. The base differentiation media consists of Iscove’s modified Dulbecco’s medium (Gibco, catalog #12440-061) supplemented with 1% l-glutamine (Gibco, catalog #25030081), 2% penicillin-streptomycin (Gibco, catalog #15140-122), human holo-transferrin (330 μg/ml; Sigma-Aldrich T4132-1G), recombinant human insulin (2 IU/ml; Sigma-Aldrich 91077C), 5% inactivated human AB pooled plasma (Rhode Island Blood Center X00004), Epogen (3 U/ml), doxycycline (1 μg/ml), and rhSCF (100 ng/ml). The supplementation of rhSCF and doxycycline to the differentiation media was stopped at days 5 and 8, respectively.

### Transduction of HUDEP-2 cells

LV1-5 lentiviral vectors (Addgene #68411) expressing either SEC23A or SEC23B fused to a C-terminal HA tag were assembled using the NEBuilder HiFi DNA assembly cloning kit (NEB). The integrity of the assembly was confirmed by Sanger sequencing. The same LV1-5 lentiviral vector expressing no protein was used as a negative control. To package the constructs into lentiviral particles, 3.56 μg of each lentiviral vector was individually cotransfected with 2.67 μg of psPAX2 (Addgene #12260, a gift from D. Trono) and 1.78 μg of pCMV-VSV-G (Addgene #8454, a gift from R. Weinberg) (*84*) into HEK293T cells using FuGENE HD Transfection Reagent (Promega). Twenty-four hours after transfection, media was changed, and subsequently, viral supernatants were collected ~12 hours afterward, centrifuged at 500*g* for 5 min, aliquoted, snap-frozen in liquid nitrogen, and stored at −80°C until use. Viral transduction in HUDEP-2 cells was performed via a 2-hour spinfection at a multiplicity of infection (MOI) of ~0.3. Twenty-four hours following spinfection, transduced cells were selected with puromycin (Sigma-Aldrich P8833) at a concentration of 1 μg/ml for 96 hours.

To activate the expression of *SEC23A*, HUDEP-2 cells were first transduced with lentiMPHv2 (lentiMPHv2 was a gift from F. Zhang, Addgene plasmid #89308) at an MOI of ~0.3. Twenty-four hours after transduction, cells were selected with hygromycin (Invitrogen #10687010) at 300 μg/ml for 15 days. Subsequently, and after a recovery period of 2 to 4 days, cells were transduced with lentiSAMv2 (lentiSAMv2 was a gift from F. Zhang, Addgene plasmid #75112) that expresses a SEC23A-targeting sgRNA or a nontargeting (NT) control sgRNA, again at an MOI of 0.3. Transduced cells were selected with blasticidin (Gibco R21001) at 8 μg/ml for 10 days and subsequently analyzed. Two independent sgRNAs targeting SEC23A (CRISPRa *SEC23A* sgRNA-1: AGAGGAGGGGACGGGGCGTG and CRISPRa *SEC23A* sgRNA-2: AAGCAAGCTCAGGGGTCCGG) and an NT sgRNA (CRISPRa NT sgRNA: CTGAAAAAGGAAGGAGTTGA) were used and cloned into lentiSAMv2 as previously described (*85*).

### Study approval

All experiments using mice were approved by and performed in accordance with the regulations of the University Committee on Use and Care of Animals.
