# Supplementary material for: SEC23A rescues SEC23B-deficient congenital dyserythropoietic anemia type II
Source: Sci Adv. 2021 Nov 24;7(48):eabj5293. doi: 10.1126/sciadv.abj5293 (PMC8612686; doi:10.1126/sciadv.abj5293)
Supplement: Supplementary file 1 — Figs. S1 to S9 [file sciadv.abj5293_sm.pdf]

Science Advances
AAAS

Supplementary Materials for

SEC23A rescues SEC23B-deficient congenital dyserythropoietic anemia type II

Richard King, Zesen Lin, Ginette Balbin-Cuesta, Gregg Myers, Ann Friedman, Guojing Zhu, Beth McGee, Thomas L. Saunders, Ryo Kurita, Yukio Nakamura, James Douglas Engel, Pavan Reddy, Rami Khoriaty*

*Corresponding author. Email: ramikhor@umich.edu

Published 24 November 2021, *Sci. Adv.* **7**, eabj5293 (2021)
DOI: 10.1126/sciadv.abj5293

This PDF file includes:

Figs. S1 to S9

Wild Type control | Erythroid *Sec23a* deletion

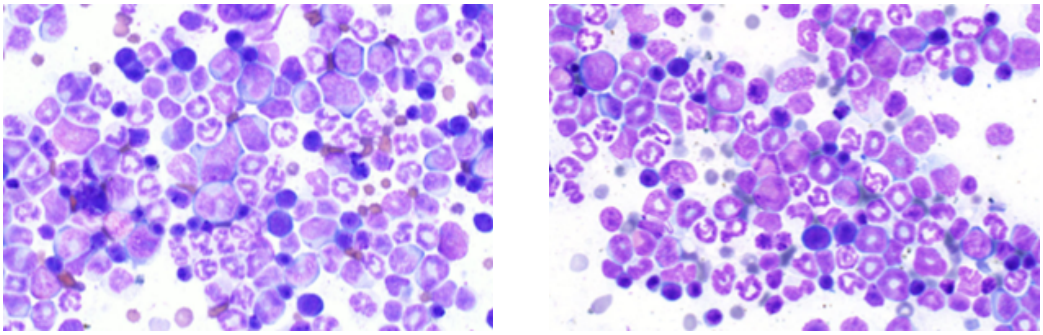

Supplemental Figure 1. Bone marrow cytology of mice with erythroid-specific SEC23A deficiency shows normal erythroid maturation and no evidence of bi- or multi-nucleated erythroblasts.

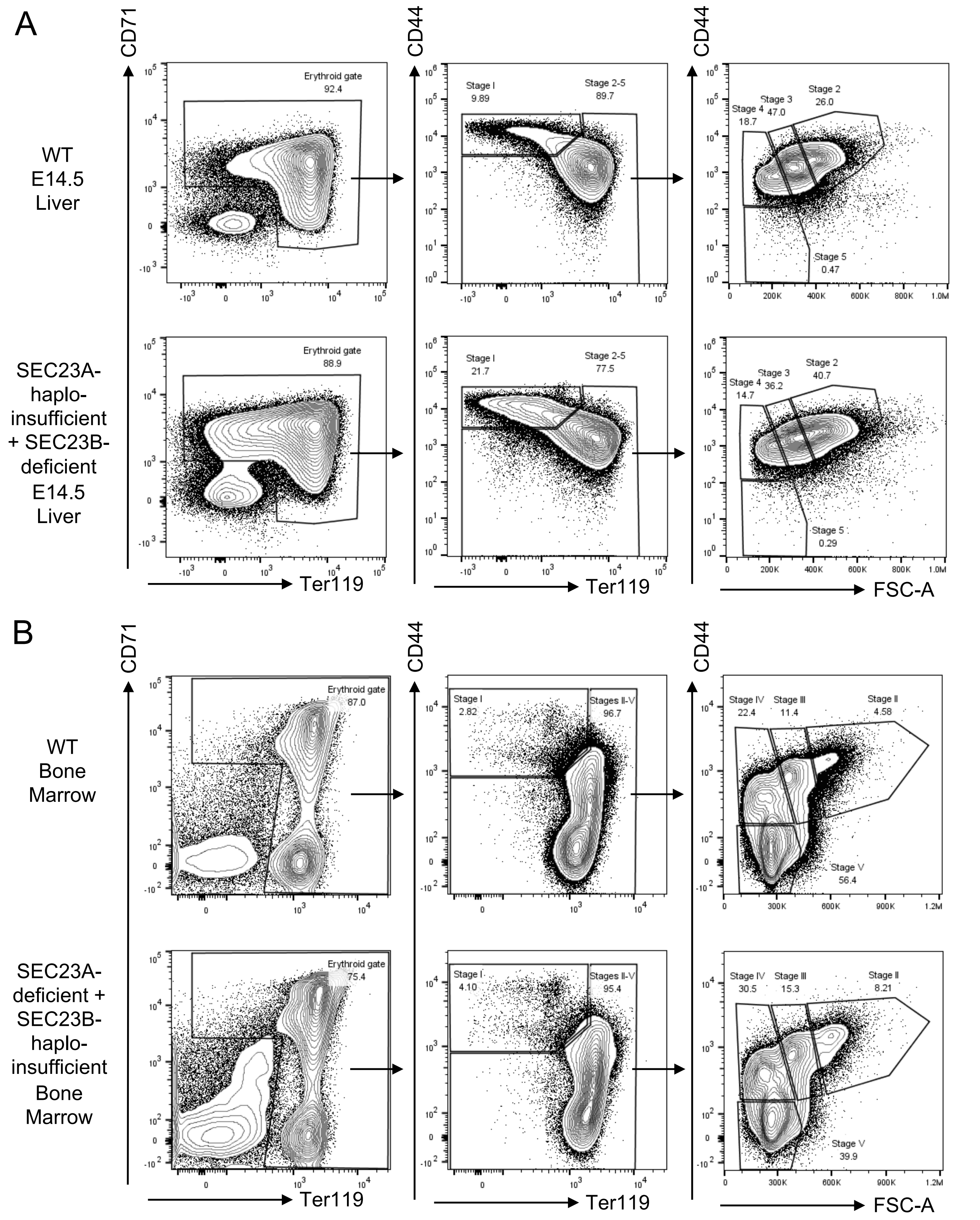

Supplemental Figure 2. Analysis of the fetal liver and adult murine bone marrow erythroid compartments. (A) E14.5 fetal liver or (B) adult murine bone marrow live, singlet, B220/GR1/CD11b-negative (not shown) cells were stratified into 5 stages of erythroid differentiation, stages I to V, as demonstrated by the gating strategy in the figure.

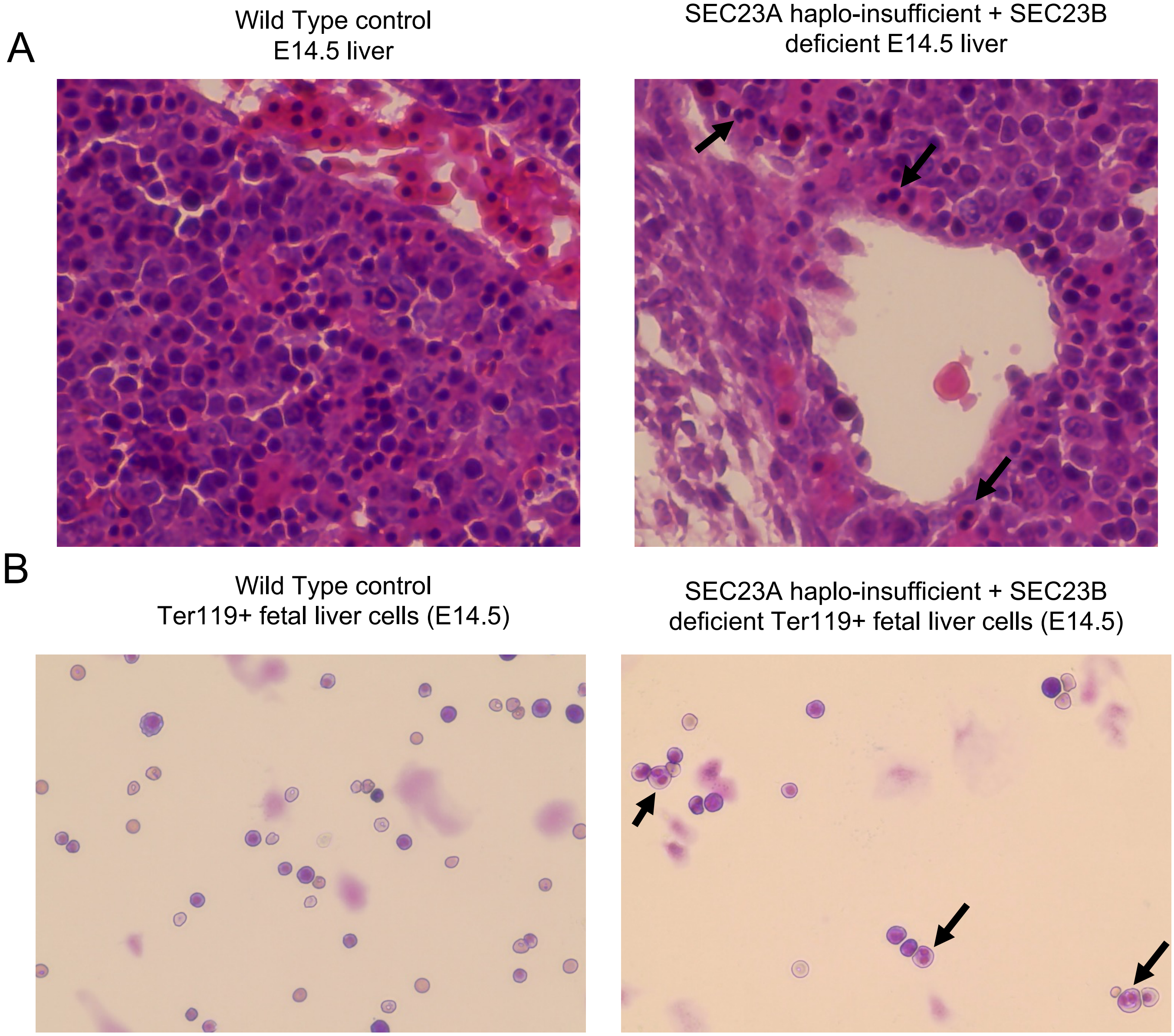

Supplemental Figure 3. E14.5 mice with erythroid-specific *Sec23a* haploinsufficiency and biallelic *Sec23b* deletion exhibit increased binucleated erythroblasts (arrows) as demonstrated by (A) analysis of liver histology and (B) morphologic evaluation of erythroblasts on cytospins of Ter119+ sorted fetal liver cells.

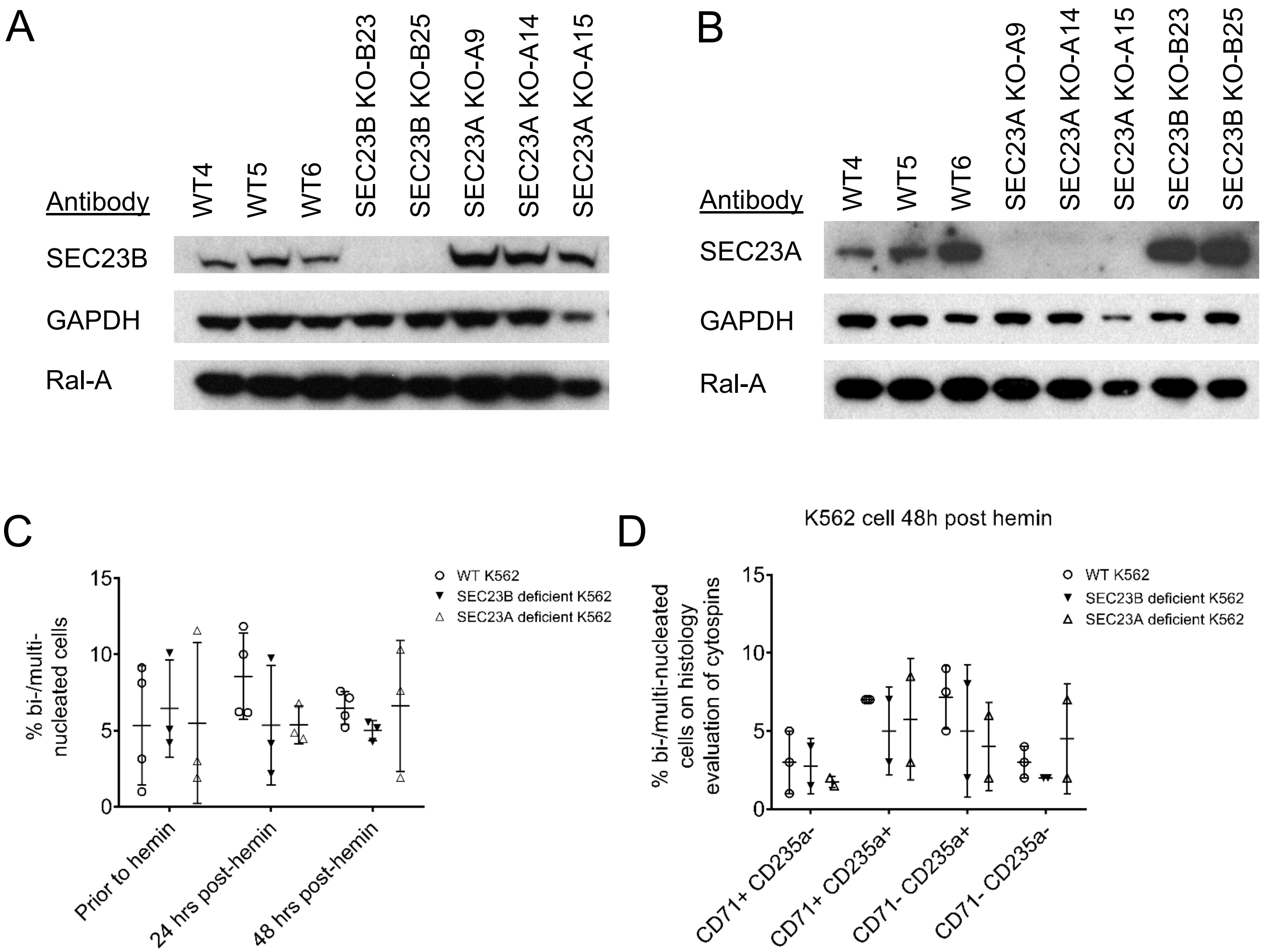

Supplemental Figure 4. SEC23A deficient and SEC23B deficient K562 cells do not exhibit bi/multi-nuclearity. (A-B) Clonal K562 cell lines with biallelic frameshift mutations in *SEC23A* or *SEC23B*, generated by transient expression of either a *SEC23A* or a *SEC23B* targeting CRISPR/Cas9 sgRNA, demonstrate absence of SEC23A or SEC23B protein, respectively, by immunoblotting. (C) Wildtype (WT), SEC23B-deficient, or SEC23A-deficient K562 cell lines were treated with hemin to induce erythroid differentiation, and bi/multi-nucleated cells were quantified using Hoechst staining at the indicated timepoints. (D) At 48 hours post-hemin treatment, WT, SEC23B deficient, or SEC23A deficient K562 cells were FACS sorted into 4 populations based on CD71 and CD235a expression as indicated. Cytospins of the sorted cell populations were reviewed and the percentages of bi/multi-nucleated cells were quantified. Two-sided, unpaired t-tests were used to compare means. P-values not significant (n.s.) if not marked. Data represent mean ± SD.

A

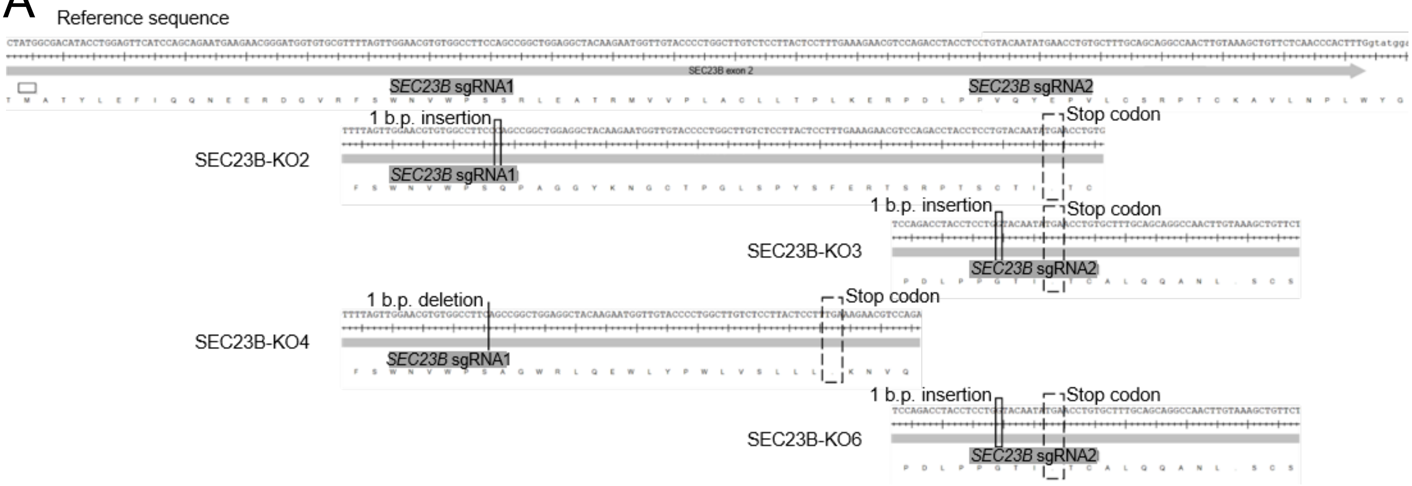

B

Plasmid	Number of sorted HUDEP-2 single cells	Number of viable clonal cell lines in expansion media (% of sorted cells)	Number of clones with bi-allelic frameshift indels (% of viable clonal lines)
PX458-23B-2.2	192	21 (11%)	4 (19%)
PX458-23B-3.1	192	31 (16%)	4 (13%)
PX458-empty	44	6 (14%)	0 (0%)

C

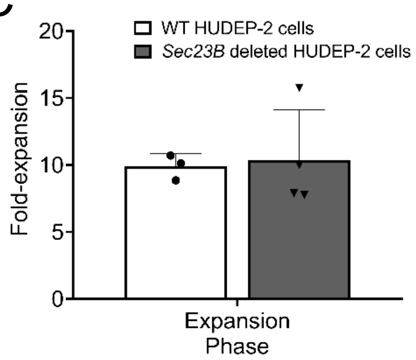

D

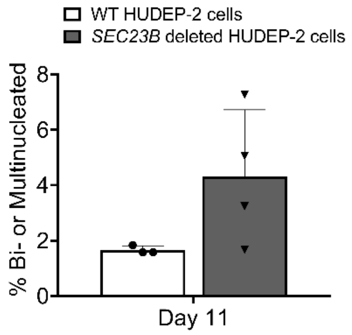

Supplemental Figure 5. Generation of clonal SEC23B-deficient HUDEP2-cell lines. (A) Two independent sgRNAs targeting *SEC23B* exon 2 were cloned in the PX458 plasmid and transiently expressed in HUDEP-2 cells. Clonal HUDEP-2 cells with biallelic frameshift mutations in *SEC23B* were generated and confirmed by PCR across the sgRNA target sites and Sanger sequencing. Indels are indicated by solid lines and premature stop codons are indicated by dashed lines. (B) Viable clonal lines were generated after FACS single cell sorting. (C) Clonal SEC23B-deficient HUDEP-2 cells expand normally in expansion media. (D) Following 11 days of differentiation, cytospins of WT and SEC23B-deficient cells were examined by an observer blinded to the genotype, and the percentages of bi- or multinucleated cells were determined. Two-sided, unpaired t-tests were used to compare means. P-values not significant (n.s.) if not marked. Data represent mean ± SD.

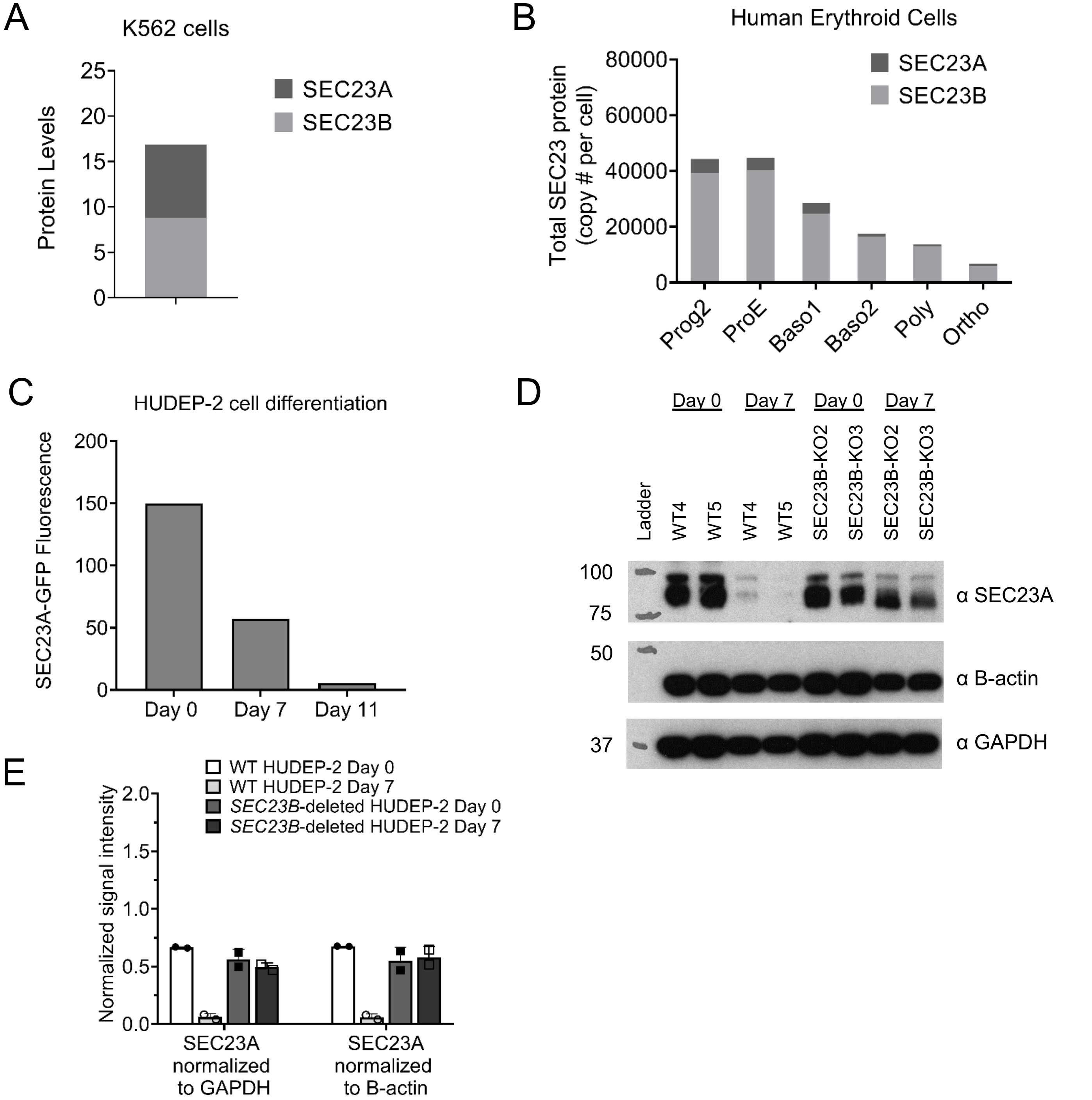

Supplemental Figure 6. SEC23A expression in K562 cells, primary human erythroid cells, and HUDEP-2 cells. (A) SEC23A and SEC23B protein levels are relatively similar in K562 cells (6). (B) The SEC23B protein is more abundant than SEC23A in human erythroid cells differentiated *in vitro* from CD34+ hematopoietic stem and progenitor cells (30). SEC23A protein is nearly absent past the basophilic erythroblast stage. (C) Wildtype reporter HUDEP-2 cells expressing GFP-fused SEC23A from the endogenous *SEC23A* locus were evaluated by flow cytometry at days 0, 7, and 11 of erythroid differentiation, and GFP fluorescence (corrected to background fluorescence) was determined. The level of SEC23A-GFP expression in HUDEP-2 cells is negligible at day 11 of differentiation, consistent with the expression of SEC23A during differentiation of human erythroid cells (see panel B). (D-E) Similarly, the SEC23A protein level in wildtype (WT) HUDEP-2 cells (measured by Western blot) decreases with differentiation, consistent with the data in panels B and C. The SEC23A protein level in SEC23B-deficient HUDEP-2 cells is comparable to that in WT HUDEP-2. The band intensities in panel D are quantified in panel E. Abbreviations: Prog2, Progenitor 2 (primarily CFU-E); ProE, proerythroblast; Baso1, basophilic erythroblast 1; Baso2, basophilic erythroblast 2; Poly, polychromatic erythroblast; Ortho, orthochromatic erythroblast. Two-sided, unpaired t-tests were used to compare means. P-values: * < 0.05, ** < 0.01. P-values not significant (n.s.) if not marked. Data represent mean ± SD.

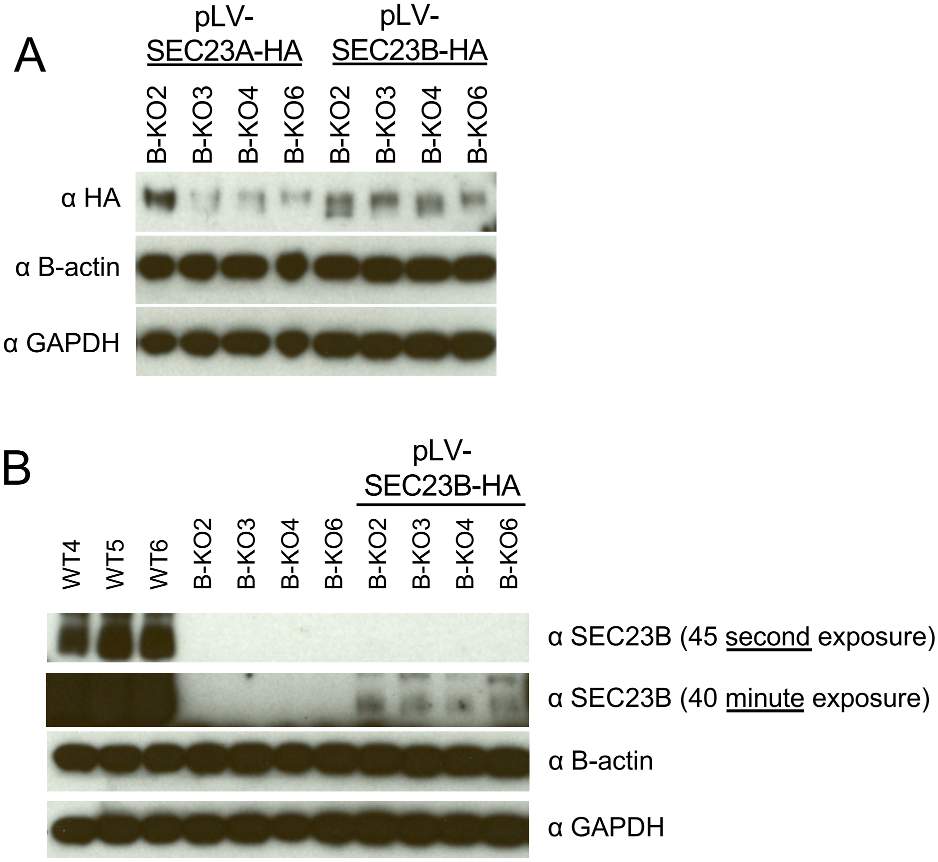

Supplemental Figure 7. Transduction of lentivirus expressing SEC23A-HA or SEC23B-HA. (A) HUDEP-2 cells were transduced with lentivirus that express SEC23A-HA, SEC23B-HA, or neither as negative control. Relatively similar levels of SEC23A and SEC23B expression were achieved, as demonstrated by immunoblotting for the HA tag. (B) SEC23B-deficient HUDEP-2 cells were transduced with lentivirus that express SEC23B-HA. The level of SEC23B protein achieved with the SEC23B-HA lentiviral construct was ~5% or less than that of the endogenous SEC23B level. Note, the image shown here is from the same western blot shown in Figure 5A, where the first 7 lanes were shown; here the last 4 lanes are also shown from cell lysates transduced with the pLV-SEC23B-HA construct.

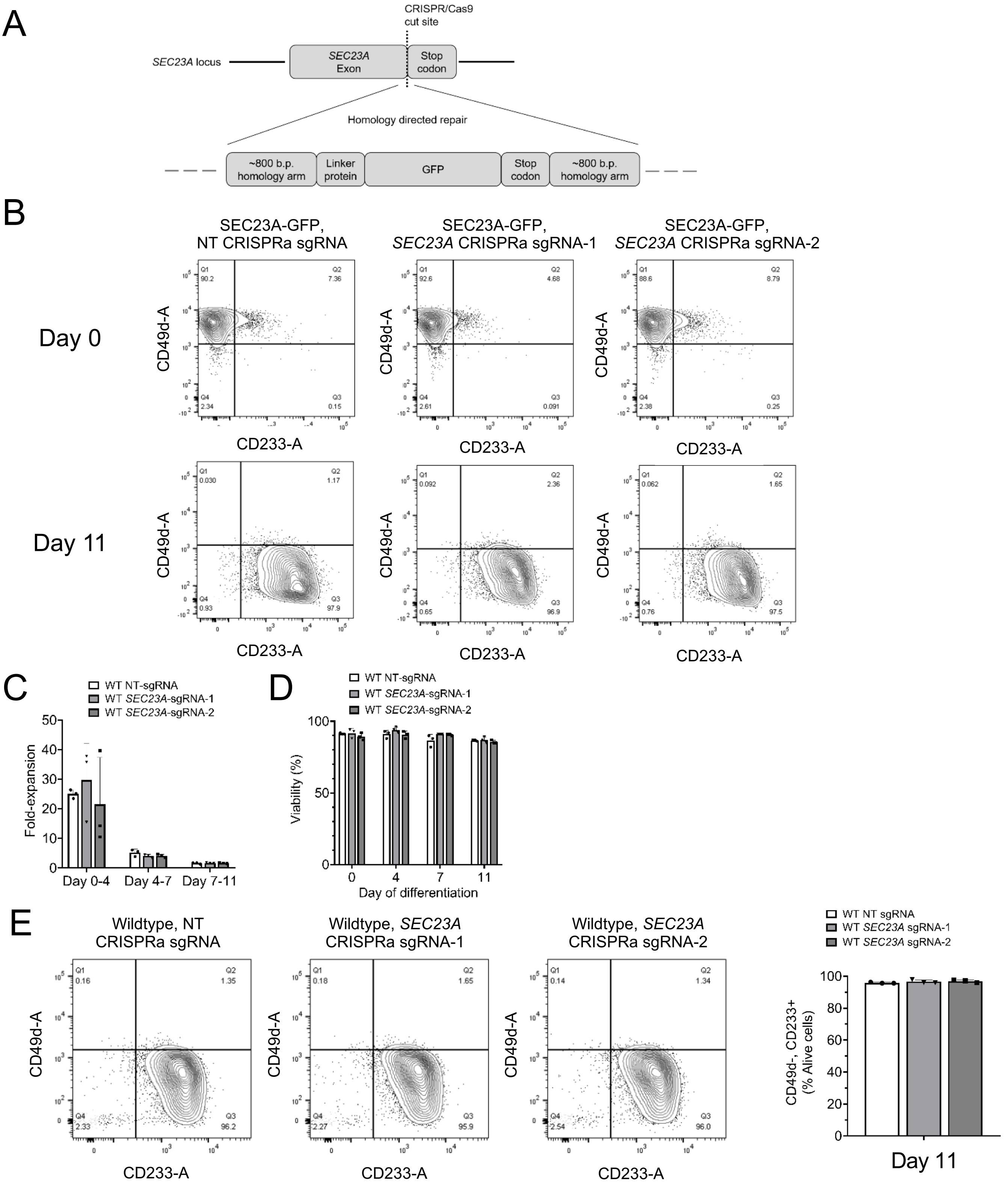

Supplemental Figure 8. Increased SEC23A expression in HUDEP2 cells does not result in a detectable erythroid defect. (A) A clonal HUDEP-2 cell line that expresses GFP-tagged SEC23A from the endogenous *SEC23A* locus was generated by transient co-expression of Cas9, an sgRNA (cloned into PX459) targeting the genomic sequence at the *SEC23A* stop codon, and a donor template for homology directed repair. (B) Wildtype reporter HUDEP-2 cells expressing GFP-tagged SEC23A were transduced with a non-targeting sgRNA or one of two CRISPRa sgRNAs targeting *SEC23A.* The reporter cell line exhibited normal erythroid maturation with or without increased SEC23A expression (n=1 for each condition). (C-E) Increased SEC23A expression in three independent wildtype HUDEP-2 clonal cell lines resulted in similar (C) fold-expansion, (D) viability, and (E) erythroid differentiation compared to cells transduced with a non-targeting sgRNA (n=3 independent clonal lines per sgRNA). Two-sided, unpaired t-tests were used to compare means. P-values not significant if not marked. Data represent mean ± SD.

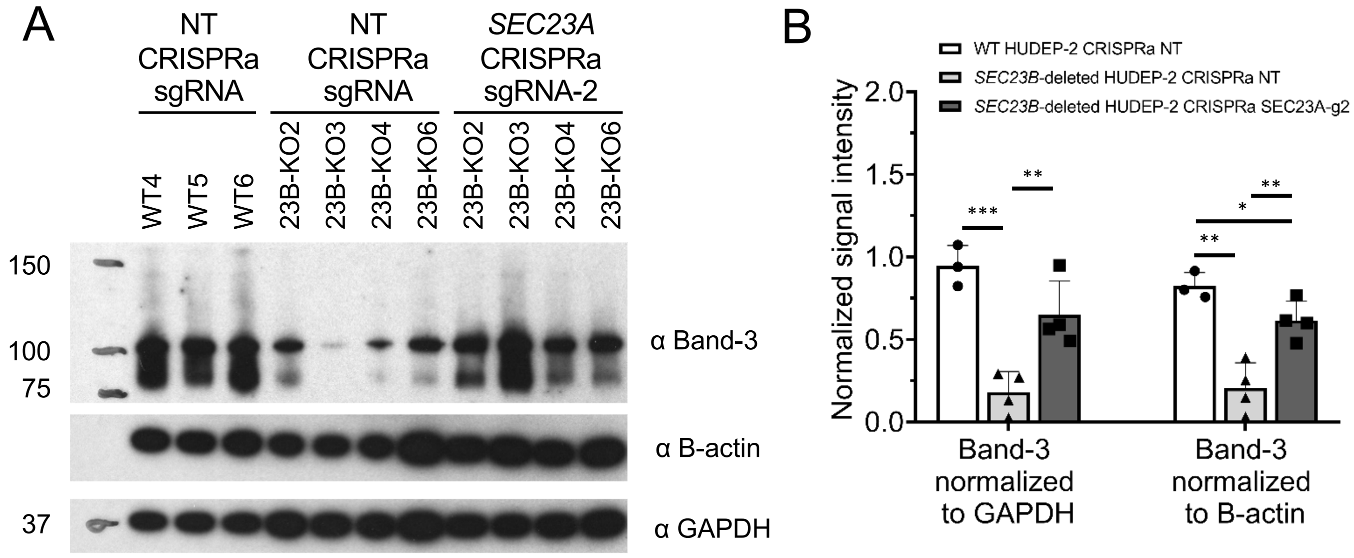

Supplemental Figure 9: SEC23B-deficient HUDEP-2 cells exhibit the characteristic CDAII band-3 defect. (A) SEC23B-deficient HUDEP-2 cells exhibit narrower size of the erythroid membrane protein band-3 (by Western blot) compared to wildtype cells at day 7 of differentiation. Increasing SEC23A expression in *SEC23B* deleted HUDEP-2 cells, using a *SEC23A*-targeting CRISPRa gRNA, restores the normal band-3 size. (B) The signal intensity of band-3 from panel A was quantified and normalized to GAPDH or B-actin. Statistical tests: Two-sided, unpaired t-tests were used to compare means. P-values: * < 0.05, ** < 0.01, *** < 0.001. P-values not significant (n.s.) unless otherwise marked. Data represent mean ± SD.
